# Supplementary material for: Energy and climate policy implications on the deployment of low-carbon ammonia technologies
Source: Nat Commun. 2025 Jan 17;16:776. doi: 10.1038/s41467-025-56006-6 (PMC11742672; doi:10.1038/s41467-025-56006-6)
Supplement: Supplementary file 1 — Supplementary Information [file 41467_2025_56006_MOESM1_ESM.pdf]

Energy and Climate Policy Implications on the Deployment of Low-Carbon Ammonia Technologies

Chi Kong Chyong[1,2], Eduardo Italiani[2], Nikolaos Kazantzis[3]

[1] Oxford Institute for Energy Studies, Oxford, OX2 6FA, UK
[2] Center on Global Energy Policy, School of International and Public Affairs, Columbia University, New York, NY 10027, USA
[3] Department of Chemical Engineering, Worcester Polytechnic Institute, Worcester, MA 01609, USA
Corresponding author: Kong.Chyong@oxfordenergy.org

Contents

Supplementary Methods **2**
Technical Methods 2
Technical Methods for AP SMR and AP CCS 3
Technical Methods for AP BH2S 5
Technical Methods for AP AEC 6
Techno-Economic Methods 8
Techno-Economic Methods for CAPEX 8
Techno-Economic Methods for OPEX 12
Economic Methods 17
Economic Methods for Discounted Cash Flow model 17
Economic Methods for Policy model 20
Economic Methods for Measurement metrics 21
Electricity Source Methods 24
Electricity Source Methods for Formulation 25
Electricity Source Methods for Implementation 28
Electricity Source Methods for Results 28
Scenario-Specific Methods 28
Scenario-Specific Methods for Scenario B 31
Scenario-Specific Methods for Power-Purchase Agreement 33
Scenario-Specific Methods for Carbon Border Adjustment Mechanism 34

Supplementary Discussion **35**
AP through Steam Methane Reforming (baseline scenario) 35
AP SMR with a Carbon Capture System (AP CCS) 36
AP SMR with a Biomass-derived feedstock (AP BH2S) 36
AP via Alkaline Electrolysis (AP AEC) 36
Economic and environmental comparison of AP across the literature 37
Haber-Bosch Flexibility 38
Convergence of Monte Carlo results 39
Comparison of SMR baseline to IEA 39
Sensitivity analysis of inputs 40
CAPEX-related sensitivities 41
OPEX-related sensitivities 41
Policy sensitivities 42
Conclusion on the sensitivity analysis 43

Supplementary Figures 44

Supplementary Tables 46

Supplementary Methods

Technical Methods

The technical underpinnings of the proposed techno-economic performance assessment framework are associated with a fixed-scale configuration of various integrated process units found in the pertinent literature as various process units illustrated in supplementary figure 1.

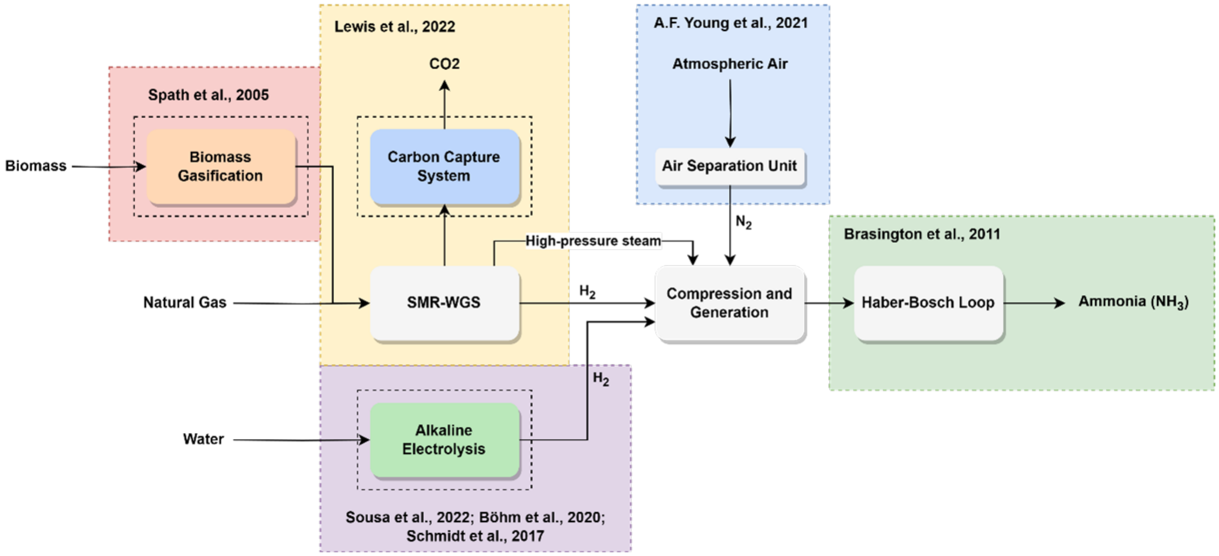

Supplementary Figure 1: Structure of composite technical process system. This figure provides a simplified illustration of the structure of a composite technical process system. Abbreviations used: SMR stands for steam-methane reforming, and WGS stands for water-gas shift.

The design concept underlying the ammonia process system under consideration is aligned with the well-known Linde Ammonia Concept (LAC)[1]. The advantage of this process design (as opposed to the Kellog-Braun approach) is that the air separation unit is separate from the SMR process, reducing the capacity requirements of the SMR equipment and thus reducing the overall cost. LAC is also easy to simulate as each process block/unit can be treated isolated.

The rest of this section is dedicated to explaining how we combined these process blocks/units in an integrated process system configuration and formed the requisite technical foundations of this study.

Supplementary Table 1: Nomenclature for supplementary technical methods.

Variable	Description	Units
η_{AEC}	Electrolysis efficiency, LHV basis	H_2 LHV
$h_{\mathrm{H_2}}^{\mathrm{LHV}}$	Hydrogen LHV	kWh per kg
$\dot{M}_{\mathrm{H_2}}$	Hydrogen production flowrate	Tonnes per day (TPD)
$\dot{M}_{\mathrm{NH_3}}$	Ammonia production flowrate	TPD
$R_{\mathrm{CO_2}}$	CCS CO_2 Capture rate	w% per w%
$h_{\mathrm{NG}}^{\mathrm{LHV}}$	Natural gas LHV	MJ per kg
$\dot{M}_{\mathrm{biomass}}$	Mass flowrate of biomass	TPD
Y_{biomass}	Kg of hydrogen per tonne of biomass	Kg H_2 per tonne biomass
$\dot{M}_{\mathrm{NG}_j}$	Energy flowrate of natural gas for technology j	MMbtu per year

Abbreviations: AEC, alkaline electrolysis cell; LHV, lower heating value; H_2, hydrogen; NH_3, ammonia; CCS, carbon capture and storage; CO_2, carbon dioxide; NG, natural gas; TPD, tonnes per day; MMbtu, million British thermal units.

Technical Methods for AP SMR and AP CCS

The hydrogen production details were based on NETL's economic analysis reports, particularly Case 1, involving a conventional SMR option, and Case 2, associated with conventional SMR with an integrated CCS system[2]. For large-scale production of N_2, cryogenic distillation is preferred. The process for the ASU was also obtained from Young et al.[3]. Finally, the ammonia synthesis loop came from NETL's report by Bransington et al., particularly Case 4, stream 38[4].

Supplementary table 2 contains all relevant inlet and outlet streams of the three LAC production blocks necessary for the design of the multi-stage compression. The resulting scale is 2717 TPD of NH_3, assuming a nitrogen conversion of 99.9% through HB[4]. The ASU study scale was increased by 6.15% (2100 TPD to 2237 TPD) to match hydrogen production in a 3:1 H_2 per N_2 (H_2 flowrate in supplementary table 2) molar ratio[2,3].

The PFD, mass, and energy information of the multi-stage compression and cooling used to integrate the SMR-WGS hydrogen and the ASU nitrogen into the Haber-Bosch loop and electricity generation from surplus steam can be found in supplementary figure 2 and supplementary table 3. All compressors are modeled as isentropic. The steam turbine was assumed to have an isentropic efficiency of 72% and the make-up compressors 85%, while the resulting work was more than 50MW. The latter is an overestimate compared to EIAGHG's report on SMR AP; hence, we assume a generation of 25MW, which closely resembles the results in the EIAGHG report[5].

AP CCS and AP AEC will not include the CAPEX of the steam turbine as surplus steam is not generated. AP SMR and AP BH2S will include the steam turbine. See the attached Excel file with the equipment list called *AP_NE_Equipment_List.xlsx*.

Supplementary Table 2: Inlet and outlet streams of selected literature processes.

	H_2 Outlet[2]	N_2 Outlet[3]	Surplus Steam from H_2[2]	NH_3 Inlet[4]
**Flowrate [TPD]**	483.02	2,100.4	18,617.0	2,632.9
**Temperature [C]**	30	40	399	21
**Pressure [MPa]**	6.48	0.0980	3.1	13.614
**Vapor Fraction**	1	1	1	1
**H_2 [mol per mol]**	0.9998	0.0000	0.0000	0.7490
**N_2 [mol per mol]**	0.0002	1.0000	0.0000	0.2500
**H_2O [mol per mol]**	0.0000	0.0000	1.0000	0.0000
**O_2 [mol per mol]**	0.0000	243 ppb	0.0000	0.0000
**Ar [mol per mol]**	0.0000	18 ppm	0.0000	0.0010
**Total [mol per mol]**	1.0000	1.0000	1.0000	1.0000

Abbreviations: H_2, hydrogen; N_2, nitrogen; NH_3, ammonia; TPD, tonnes per day; C, degrees Celsius; MPa, megapascal; ppb, parts per billion; ppm, parts per million.

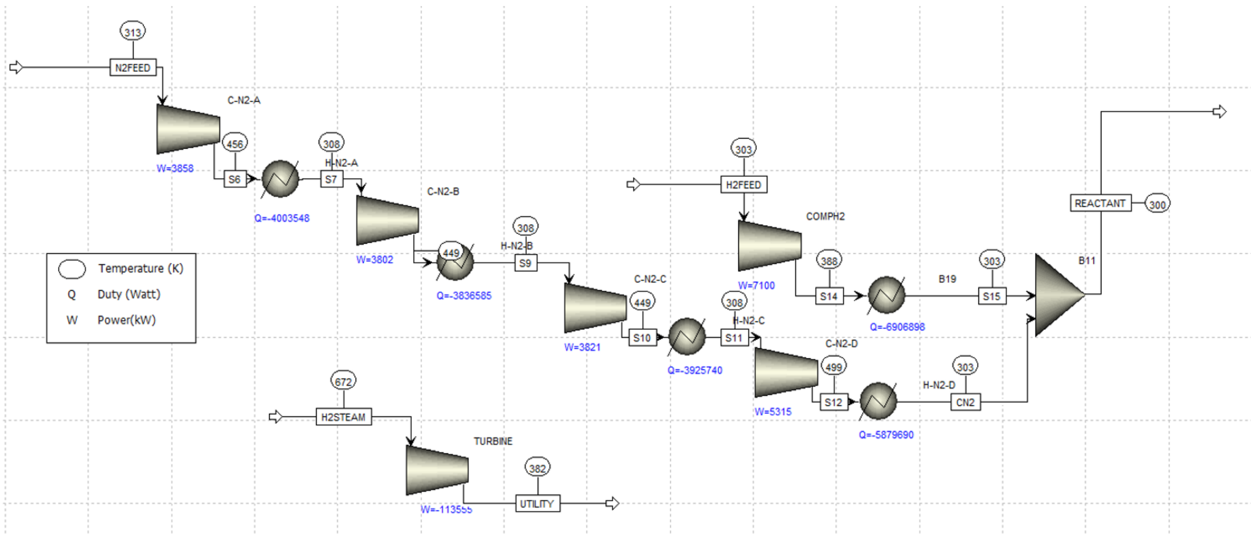

Supplementary Figure 2: Compression system for ammonia technologies. The figure illustrates the compression systems for different ammonia production technologies: AP CCS (Ammonia Plant with Carbon Capture System), AP BH2S (Ammonia Plant using Biomass Gasification coupled with Steam Methane Reforming), and AP SMR (Ammonia Plant using Steam Methane Reforming). Note that AP CCS does not generate electricity.

Supplementary Table 3: Stream information for make-up compression and steam turbine for AP SMR, AP CCS, and AP BH_2S.

	H_2FEED	N_2FEED	REACTANT	STEAM IN	STEAM OUT	NH_3˙OUT
**Flowrate [TPD]**	483.0	2237.0	2720.0	18617.0	18617.0	2717.0
**Flowrate [kmol per hr]**	9958.0	3327.3	13285.3	43058.4	43058.4	6647.4
**Temperature [C]**	30	40	26.6	399	109.3	21
**Pressure [MPa]**	6.48	0.098	13.614	3.1	0.1	13.614
**Vapor Fraction**	1	1	1	1	1	1
**H_2 [mol per mol]**	1.000	0.000	0.749	0.000	0.000	0.000
**N_2 [mol per mol]**	0.000	1.000	0.251	0.000	0.000	0.000
**H_2O [mol per mol]**	0.000	0.000	0.000	1.000	1.000	0.000
**O_2 [mol per mol]**	0.000	0.000	0.000	0.000	0.000	0.000
**Ar [mol per mol]**	0.000	0.000	0.000	0.000	0.000	0.000
**NH_3 [mol per mol]**	0.000	0.000	0.000	0.000	0.000	1.000
**Total [mol per mol]**	1.000	1.000	1.000	1.000	1.000	1.000

Abbreviations: H_2, hydrogen; N_2, nitrogen; NH_3, ammonia; SMR, steam methane reforming; CCS, carbon capture and storage; BH_2S, blue hydrogen sulfide; TPD, tonnes per day; kmol per hr, kilomoles per hour; C, degrees Celsius; MPa, megapascal.

Technical Methods for AP BH2S

The AP BH2S unit was obtained from Spath et al. and was combined with the inlet of the SMR-WGS hydrogen production process[6]. Specifically, stream 327 of the current design for BH2S was combined with stream 3 of Case 1 of Lewis et al.[2,6]. Table A 3 includes the difference in the stream compositions before entering the SMR reactor. The SMR reactors for both studies are modeled as equilibrium reactors with similar design parameters (S/C ratio, pressure, and temperature)[2,6]. Hence, for the BH2S scenario, we assume that the SMR outlet composition, temperature, and pressure differences are negligible for this techno-economic analysis. Given this assumption, we assume the process equipment before the SMR reactor comes from Spath et al.[6]. After the SMR reactor, we assume the equipment from Lewis et al.[2] is used. To ascertain the amount of biomass required to fulfill the hydrogen production of the SMR process in Lewis et al.[2], we use the hydrogen yield from dry biomass by Spath et al.[6]. Calculation formula found below in supplementary equation (1).

$$M_{\text{biomass}} = M_{\text{H}_2} \times 1000 \times \frac{1}{Y_{\text{biomass}}} \quad (1)$$

Technical Methods for AP AEC

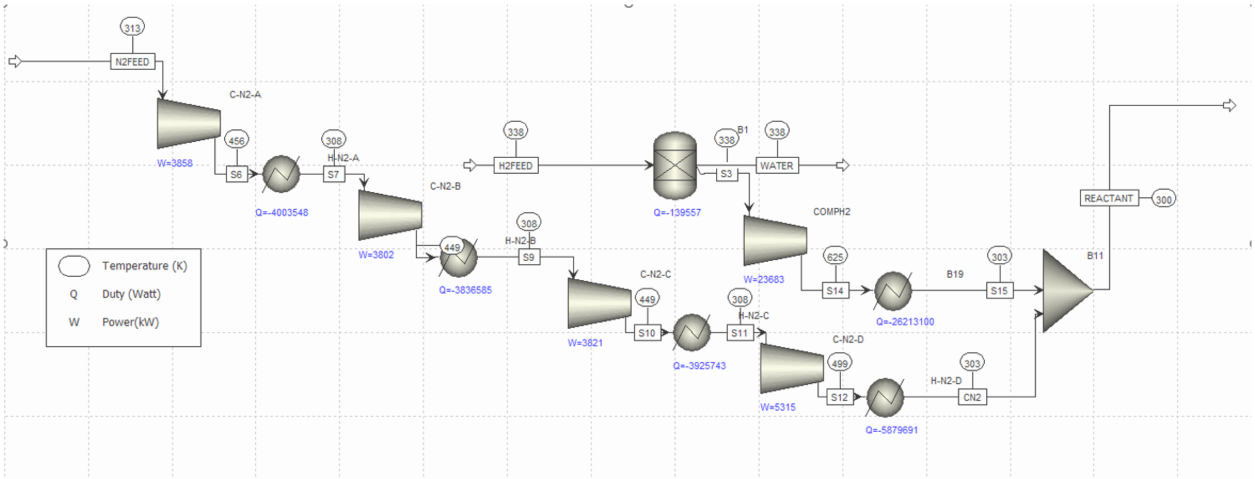

Supplementary Figure 3: Compression system for ammonia technologies. This figure illustrates the compression system for ammonia production through alkaline electrolysis. Abbreviations used: AEC stands for Alkaline Electrolysis Cell.

For the electrolysis system, the CAPEX and OPEX values were obtained from the literature[7,8]. A robust study by Sousa and colleagues estimated the outlet conditions of the electrolysis hydrogen stream – their values were used to model the inlet in the compression module[9]. We acknowledge that Sousa and colleagues prefer PEM, not AEC, as the technology option. Nevertheless, this assumption is appropriate. The outlet hydrogen stream from PEM and AEC are found under similar conditions. Schmidt et al. indicate that AEC H_2 outlet conditions are less than 30 bar and 60-80 C^o. Sousa et al.'s H_2 outlet conditions are 29 bar and 65 C^o; therefore, this assumption holds[8,9]. Hence, the only difference between AEC and PEM using our modeling methodology would be the cost per kW of capacity, energy efficiency, and cell lifetime. We tune these parameters to AP AEC (see supplementary economic methods).

The water content in the hydrogen stream is separated by a separator block in ASPEN and is cost-estimated as a cylindrical vertical vessel (2m diameter by 10m length) using the heuristics proposed by Turton et al.[10] (see Excel file named *AP_NE_Equipment_List.xlsx*).

Supplementary Table 4: BH_2S composition difference between Lewis et al. (2022) and Spath et al. (2005) at the SMR inlet.

**Species**	**Xi AP**	**Xi AP BH_2S**
H_2O	67.6%	40%
H_2	4.3%	32%
CO	0%	19%
CO_2	1.7%	8%
CH_4	25.8%	1%

Abbreviations: H_2O, water; H_2, hydrogen; CO, carbon monoxide; CO_2, carbon dioxide; CH_4, methane; Xi AP, species composition from Lewis et al. (2022); Xi AP BH_2S, species composition from Spath et al. (2005).

Supplementary Table 5: Key technical parameters.

Input Name	Value	Unit	Source
$\dot{M}_{NH_3}$	2717	TPD	Calculated assuming 99.9% conversion of N_2
$\dot{M}_{H_2}$	483.013	TPD	[2]
η_{AEC}	uniform(70%, 78%)	-	[11]
$h^{LHV}_{H_2}$	33.3333	kWh per kg	
R_{CO_2}	95.6%	W%/w%	[2]
h^{LHV}_{NG}	47.1	MJ per kg	[2]
$Y_{biomass}$	70.1	Kg H_2 per tonne biomass	[6]

Abbreviations: $\dot{M}_{NH_3}$, ammonia production flowrate; $\dot{M}_{H_2}$, hydrogen production flowrate; η_{AEC}, alkaline electrolysis cell efficiency; $h^{LHV}_{H_2}$, lower heating value of hydrogen; R_{CO_2}, carbon capture and storage CO_2 capture rate; h^{LHV}_{NG}, lower heating value of natural gas; $Y_{biomass}$, hydrogen yield from biomass; TPD, tonnes per day; W%/w%, weight percent; MJ, megajoule; kWh, kilowatt-hour.

Supplementary Table 6: Electricity requirements.

Input Name	Value	Unit	Source
**AP SMR**	HP: 13, AP: 64	MW	[2–4,6]
**AP CCS**	HP: 41, AP: 117	MW	
**AP BH$_2$S**	HP: 76.6, AP: 127	MW	
**AP AEC**	HP: $\frac{\dot{M}_{H_2} \times h^{LHV}_{H_2} \times 1000}{\eta_{AEC}}$, AP: $\frac{\dot{M}_{H_2} \times h^{LHV}_{H_2} \times 1000}{\eta_{AEC}} + 52$	MW	[2–4,7]

HP stands for hydrogen production and shows the electricity demand for hydrogen production. AP is ammonia production including hydrogen production, Haber-Bosch (HB) process, air separation unit (ASU), and compression system. Abbreviations: MW, megawatt; $\dot{M}_{H_2}$, hydrogen production flowrate; $h^{LHV}_{H_2}$, lower heating value of hydrogen; η_{AEC}, alkaline electrolysis cell efficiency.

Supplementary Table 7: Natural gas requirements, M_{NG_j}.

Input Name	Value	Unit	Source
**AP SMR**	25,039,232.02	MMBtu per yr	[2]
**AP CCS**	26,624,225.74	MMBtu per yr	[2]
**AP BH$_2$S**	0	MMBtu per yr	Assumption
**AP AEC**	0	MMBtu per yr	Assumption

The natural gas flowrates were directly obtained from case 1 and 2 of Lewis et al.[2]. Refer to stream number 3. We used the h^{LHV}_{NG} and the MMBtu conversion factor to convert to energy units. Abbreviations: M_{NG_j}, natural gas energy flowrate for technology j; AP, ammonia production; SMR, steam methane reforming; CCS, carbon capture and storage; BH$_2$S, blue hydrogen sulfide; AEC, alkaline electrolysis cell; MMBtu, million British thermal units; h^{LHV}_{NG}, lower heating value of natural gas.

Supplementary Table 8: Water demand.

Input Name	Value	Unit	Source
**AP SMR**	52,709,532,285	Kg per year	[2–4,6]
**AP CCS**	54,034,044,285	Kg per year	
**AP BH_2S**	53,448,253,002	Kg per year	
**AP AEC Osmosis**[A]	1,332,790,200, 73,077,302,619	Kg per year	[2–4,9]

[A] The first value provided is the reverse osmosis flowrate. The second value is the process water demand. For more details on how the water demand was calculated, see the annexed Excel file called `AP_NE_Water_Balance.xlsx`. Abbreviations: AP, ammonia production; SMR, steam methane reforming; CCS, carbon capture and storage; BH_2S, blue hydrogen sulfide; AEC, alkaline electrolysis cell; Kg, kilogram.

Techno-Economic Methods

This section aims to determine the equipment capacities for the AP process by combining the processes discussed in the previous section. These equipment capacities directly influence the capital expenditure ($\mathrm{CAPEX_j}$) associated with technology j, as defined in supplementary equation (2):

$$j \in \{\text{AP SMR}, \text{AP CCS}, \text{AP BH}_2\text{S}, \text{AP AEC}\} \tag{2}$$

Additionally, the demands for feedstock, utilities, and other raw materials can be obtained from the mass and energy balances from the reports. This will constitute the variable operating costs. The fixed operating costs, however, are estimated based on the number of unit operations required to operate the plant. These types of costs constitute the operational expenditure ($\mathrm{OPEX_j}$).

The goal of this section is to describe the methodology used to estimate the $\mathrm{CAPEX_j}$ and $\mathrm{OPEX_j}$ for each technology option, following the techno-economic approach outlined by Peters et al.[12]. These two quantities will allow for calculating the desired financial variables in ensuing sections (i.e., NPV, CAC, etc.).

Techno-Economic Methods for CAPEX

The full nomenclature for CAPEX variables can be found in supplementary table 9. Values for heuristics used to arrive at the CAPEX are in supplementary table 12. Installed and uninstalled costs can be found in supplementary table 11.

Supplementary Table 9: Nomenclature for the CAPEX section.

Parameter	Description	Units
j	Technology index	
z	Cost factor index	
x_z	CAPEX cost factor	% EC_{u_j}
$CAPEX_j$	Capital Expenditure cost for technology j	\$
$P_{(n_0,t_0,z)}$	Base capacity equipment cost	\$
m_n	Number of spare equipment units required	N
S_n	Required equipment capacity	
S_{n0}	Base capacity	
i	Installation cost factor	
f	Equipment economies of scale factor [12]	
C_{t_0}	Chemical engineering plant index at t_0	
C_{2023}	Chemical engineering plant index in 2023	
M_{NG}	Energy flowrate of natural gas	MMBtu per year
M_{EL}	Electricity demand	kWh per year
$C_{\mathrm{EL}}(T)$	Electricity operating hour	hour
$CAPEX_{\mathrm{updated}_j}$	Updated CAPEX after adding the additional cost	\$
FCI_j	Fixed capital investment of technology j	\$
EC_{u_j}	Uninstalled cost for technology j	\$
EC_{i_j}	Installed cost for technology j	\$
WC_j	Working capital for technology j	\$
$C_{\mathrm{I\&C}}$	Cost factor for instrumentation and controls	% EC_{u_j}
C_p	Cost factor for piping	% EC_{u_j}
C_{el}	Cost factor for electrical	% EC_{u_j}
C_b	Cost factor for buildings	% EC_{u_j}
$C_{\mathrm{sf\&yi}}$	Cost factor for service facilities and yard improvements	% EC_{u_j}
C_{land}	Cost factor for land	% EC_{u_j}
C_{eng}	Cost factor for engineering	% EC_{u_j}
C_L	Cost factor for legal	\$
C_c	Cost factor for construction	% EC_{u_j}
C_{co}	Cost factor for contingency	% EC_{u_j}
C_{WC}	Cost factor for working capital	% FCI
C_{AEC}	Cost of AEC electrolyzer	\$ per kWe

Abbreviations: CAPEX, capital expenditure; EC_{u_j}, uninstalled cost for technology j; FCI, fixed capital investment; MMBtu, million British thermal units; kWh, kilowatt-hour; AEC, alkaline electrolysis cell. The symbols and parameters represent various cost factors and indices used in the CAPEX calculation.

Supplementary Table 10: Values for x_z excluding WC_j.

Input Name	Value	Unit	Source
$C_{\text{I\&C}}$	uniform(0.08, 0.55)	% Purchased equipment (PE)	[12]
C_p	uniform(0.1, 0.8)	% PE	[12]
C_{el}	uniform(0.1, 0.4)	% PE	[12]
C_b	uniform(0.1, 0.7)	% PE	[12]
$C_{\text{sf\&yi}}$	uniform(0.05, 0.18)	% PE	[12]
C_{land}	900,000	$	[2]
C_{eng}	uniform(0.05, 0.3)	% PE	[12]
C_L	uniform(0.03, 0.05)	% PE	[12]
C_c	uniform(0.3, 0.4)	% PE	[12]
C_{co}	uniform(0.35, 0.45)	% PE	[12]
C_{WC}	uniform(0.1, 0.2)	% FCI	[12]
C_{AEC}	2026: uniform(750, 1000), 2033: uniform(500, 1000)	$ per kWe	[7,8,13]

Abbreviations: PE, purchased equipment; FCI, fixed capital investment; AEC, alkaline electrolysis cell; kWe, kilowatt of electrical capacity. Values for C_{AEC} are projected for 2026 and 2033, reflecting expected cost reductions over time.

Supplementary Table 11: EC_{u_j} and EC_{i_j} for each technology.

Input Name	Value	Unit	Source
**AP SMR**	(726,947,344.6, 508,096,133.6)	$	Calculated
**AP CCS**	(1,103,130,442, 781,847,023.7)	$	Calculated
**AP BH$_2$S**	(940,144,529.3, 5,080,528,806,470)	$	Calculated
**AP AEC**	(567,914,373, 402,707,562.8)	$	Calculated

The first value in the parenthesis is EC_{i_j} and the second is EC_{u_j}. Obtained from the Excel file called `AP_NE_Equipment_List.xlsx`. Abbreviations: AP, ammonia production; SMR, steam methane reforming; CCS, carbon capture and storage; BH$_2$S, blue hydrogen sulfide; AEC, alkaline electrolysis cell; EC_{i_j}, installed cost for technology j; EC_{u_j}, uninstalled cost for technology j.

Supplementary Table 12: Values for x_z excluding WC_j.

Input Name	Value	Unit	Source
$C_{\text{I\&C}}$	uniform(0.08, 0.55)	% Purchased equipment (PE)	[12]
C_p	uniform(0.1, 0.8)	% PE	[12]
C_{el}	uniform(0.1, 0.4)	% PE	[12]
C_b	uniform(0.1, 0.7)	% PE	[12]
$C_{\text{sf\&yi}}$	uniform(0.05, 0.18)	% PE	[12]
C_{land}	900,000	$	[2]
C_{eng}	uniform(0.05, 0.3)	% PE	[12]
C_L	uniform(0.03, 0.05)	% PE	[12]
C_c	uniform(0.3, 0.4)	% PE	[12]
C_{co}	uniform(0.35, 0.45)	% PE	[12]
C_{WC}	uniform(0.1, 0.2)	% FCI	[12]
C_{AEC}	2026: uniform(750, 1000), 2033: uniform(500, 1000)	$ per kWe	[7,8,13]

Abbreviations: PE, purchased equipment; FCI, fixed capital investment; AEC, alkaline electrolysis cell; kWe, kilowatt of electrical capacity. Values for C_{AEC} are projected for 2026 and 2033, reflecting expected cost reductions over time. The parameter C_{WC} represents the working capital cost factor as a percentage of FCI.

$$\text{CAPEX}_j = \sum_{z=0}^{z} \left[(s_f \times x_z) \times \text{EC}_{u_j} \right] + \text{EC}_{i_j} + C_{\text{land}} + \text{WC}_j \tag{3}$$

Supplementary equation (3) describes how the CAPEX_j was estimated. This method obtains the installed ($\text{EC}_\text{i\_j}$) and uninstalled ($\text{EC}_\text{u\_j}$) costs for a specific technology and calculates the CAPEX_j. The values for $\text{EC}_\text{i\_j}$ and $\text{EC}_\text{u\_j}$ were calculated based on the equipment data sets described in section A. x_z is a cost factor for a set of facilities, z [12]. C_land is the cost of land and a constant for all technologies. WC_j is the working capital (calculated in supplementary equation (5)).

The average value of the cost factors for each technology for each scenario is in the annexed Excel file named *AP_NE_CAPEX.xlsx*. More detailed information on the CAPEX estimation methodology is in Peters et al. [12]. For clarity, we specify the cost factors below.

$$x_z \in \{C_{\text{I\&C}}, C_p, C_{\text{el}}, C_b, C_{\text{sf\&yi}}, C_{\text{eng}}, C_L, C_c, C_{\text{co}}\} \tag{4}$$

where each cost factor, C_z, corresponds to instrumentation and control, piping, electrical, buildings, service facilities and yard improvements, engineering, legal, construction, and contingency, respectively.

The working capital, WC_j, was calculated using the formula below (supplementary equation (5)).

$$\text{WC}_j = \text{FCI}_j \times C_{\text{WC}} = \left(\sum_{z=0}^{z} [s_f \times x_z] \times \text{EC}_{u_j} + \text{EC}_{i_j} + C_{\text{land}} \right) \times C_{\text{WC}} \tag{5}$$

The textbook cost factors used to calculate CAPEX_j and related parameters are based on a scale of the order of 100 TPD [12]. Hence, each parameter was scaled using the six-tenths rule to the AP scale of $M_{\text{NH`3}}$ (2717 TPD NH_3). The scaling factor is defined as s_f.

$$s_f = \left(\frac{1}{100/M_{\text{NH}_3}} \right)^{0.6} \tag{6}$$

$\text{EC}_\text{u\_j}$ and $\text{EC}_\text{i\_j}$ were calculated using supplementary equations (7) and (8).

$$\text{EC}_{u_j} = \sum_{n=0}^{N} \left[m_n \times P_{(n,t_0,j)} \times \left(\frac{S_n}{S_{n0}} \right)^f \times \left(\frac{C_{2023}}{C_{t_0}} \right) \right] \tag{7}$$

$$\text{EC}_{i_j} = \sum_{n=0}^{N} \left[m_n \times P_{(n_0,t_0,j)} \times \left(\frac{S_n}{S_{n0}} \right)^f \times (1+i) \times \left(\frac{C_{2023}}{C_{t_0}} \right) \right] \tag{8}$$

$\text{EC}_\text{u\_j}$ and $\text{EC}_\text{i\_j}$ are functions of the sum of the base-capacity uninstalled equipment costs $P_{(\text{n`0,t`0,z})}$ where n_0 denotes the name of the piece of equipment (see equipment list, N, in Excel file named *AP_NE_Equipment_List.xlsx*), and t_0 is the time when the equipment cost was estimated. S_n is the required capacity of the piece of equipment. S_n0 is the base equipment capacity. f is the economies of scale factor obtained from Peters et al. [12] (also in the Excel file). $(1 + i)$ is the installation factor obtained from Peters et al. [12]. C_{2023} is the chemical engineering plant index (CEPCI) in 2023 and $C_{(\text{t`0})}$ is the CEPCI at the time the piece of equipment was cost estimated. m_n is the number of spares for a given piece of equipment ($m_n \in N$).

There are times when we need to add the cost of certain modules directly into the CAPEX_j from the scientific literature. Once we do this, we also need to scale the cost components of the CAPEX $(s_f \times x_z) \times (EC_\text{u\_j})$ to be adjusted to the higher CAPEX. We perform the method described below for the AP AEC electrolyzer, solar, wind, and battery storage costs.

We define a new variable R_j to represent the scaling ratio by which to increase each CAPEX component.

$$R_j = 1 + \frac{C_{\text{additional}_j}}{\text{CAPEX}_{\text{Updated}_j}} \tag{9}$$

$\text{CAPEX}_\text{updated\_j}$ is the updated CAPEX after adding the additional cost $C_\text{additional\_j}$. This ensures that the overall CAPEX estimate accurately reflects the depreciation effect of the additional costs on the project's financial analysis.

$$\left|s_f \times x_z \times \mathrm{EC}_{u_j}\right|_{\mathrm{updated}} = \left(s_f \times x_z \times \mathrm{EC}_{u_j}\right) \times R_j \tag{10}$$

$$\mathrm{CAPEX}_{\mathrm{updated}} = \mathrm{CAPEX} + C_{\mathrm{additional}} \tag{11}$$

Specific to AP AEC, we calculate the CAPEX based on established heuristics by the literature and the electricity demand required to electrolyze water.

$$C_{\mathrm{additional}_{\mathrm{AP\ AEC}}} = \left[\frac{\dot{M}_{\mathrm{H_2}} \times h_{\mathrm{H_2}}^{\mathrm{LHV}} \times 1000}{\eta_{\mathrm{AEC}}}\right] \times C_{\mathrm{AEC}} \tag{12}$$

Techno-Economic Methods for OPEX

A full nomenclature table for the OPEX calculations can be found in supplementary table 13. Tables containing the values used for these variables are in supplementary tables 14, 15, 16, 17, 18.

Supplementary Table 13: Nomenclature for the OPEX section.

Parameter	Description	Units
$MDOPEX_j$	Market dependent OPEX for technology j	\$ per month
$MIOPEX_j$	Market independent OPEX for technology j	\$ per month
H_{shift}	Hours per shift	Hour per shift
H_{dp}	Hours per day per processing step	Hours per day per processing step
P_s	Processing steps	Processing step
F_a	Availability factor	%
$wage_h$	Operator salary	\$ per hr
L_c	Monthly operator labor cost	\$ per month
TL_{c_j}	Total labor costs	\$ per month
TF_{c_j}	Fixed charges	\$ per month
FC_j	Fixed cost	\$ per month
C_{sup}	Supervision costs to calculate total labor costs	% L_c
C_{os}	Operating supply costs to calculate total labor costs	% $FCI_j \times C_{\text{MT}}/12$
C_{LC}	Laboratory charges to calculate total labor costs	% L_c
$C_{\text{P\&R}}$	Patent and royalty costs to calculate total labor costs	% $CAPEX_j$
C_{OH}	Overhead costs to calculate total labor costs	% maintenance + supervision + labor
C_{fin}	Financing fixed cost	% $CAPEX_j$
C_{rent}	Rent fixed cost	% C_{land}
C_{ptax}	Local property tax fixed cost	% TL_c
C_{admin}	Administrative fixed cost	% C_{admin}
C_{ins}	Insurance costs	% FCI_j
C_{MT}	Maintenance costs to calculate total labor costs	% FCI_j
VC_j	Variable cost	\$ per month
$S_{\text{H}_2\text{O}}$	Water cost	\$ per kg
$M_{\text{H}_2\text{O}}$	Process water demand per month	Kg per month
$Misc_j$	Miscellaneous costs in calculating OPEX	\$ per month
$M_{\text{H}_2\text{O}}^{\text{osmosis}}$	Feedstock water demand	\$ per kg
$S_{\text{H}_2\text{O}}^{\text{osmosis}}$	Reverse Osmosis water cost	Kg per month
i	Commodity index	
$P_i(T)$	GBM price function for commodity i	\$ per unit i
$P_i(T=0)$	Initial price of commodity i	\$ per unit i
μ_i	Drift for commodity i	%
σ_i	Volatility for commodity i	%
F_{DM}	Distribution and marketing costs	\$
F_{RD}	R&D costs	\$
M_{NG}	Energy flowrate of natural gas	mmBTU per year
M_{EL}	Electricity demand	kWh per year
$OPEX_j$	Operational Expenditure for technology j	\$

Abbreviations: OPEX, operational expenditure; MDOPEX, market-dependent OPEX; MIOPEX, market-independent OPEX; FCI, fixed capital investment; CAPEX, capital expenditure; GBM, geometric Brownian motion; $P_i(T)$, price function for commodity i; M_{NG}, natural gas energy flowrate; M_{EL}, electricity demand; $M_{\text{H}_2\text{O}}$, process water demand; $S_{\text{H}_2\text{O}}$, water cost; $M_{\text{H}_2\text{O}}^{\text{osmosis}}$, feedstock water demand; $S_{\text{H}_2\text{O}}^{\text{osmosis}}$, reverse osmosis water cost; VC, variable cost; TL, total labor; R&D, research and development; mmBTU, million British thermal units; kWh, kilowatt-hour.

Supplementary Table 14: Number of processing steps by technology.

Input Name	Value
**AP SMR**	30
**AP CCS**	33
**AP BH_2S**	38
**AP AEC**	23
H_{dp}	55

Processing steps of each AP process. Used for labor cost calculations. All values are assumptions estimated from the process flow diagrams of Lewis et al., Spath et al., Brasington et al., and Young et al.[2–4,6]. Abbreviations: AP, ammonia production; SMR, steam methane reforming; CCS, carbon capture and storage; BH_2S, blue hydrogen sulfide; AEC, alkaline electrolysis cell; H_{dp}, hours per day per processing step.

Supplementary Table 15: Heuristics factors for OPEX.

Input Name	Value	Unit	Source
C_{MT}	0.0286	% $CAPEX_j$	12
C_{sup}	0.15	% L_c	12
C_{os}	0.1	% $FCI_j \times C_{\mathrm{MT}}/12$	12
C_{LC}	0.15	% L_c	12
$C_{\mathrm{P\&R}}$	0.005	% $CAPEX_j$	12
C_{OH}	0.70	% maintenance + supervision + labor	12
C_{fin}	0	% $CAPEX_j$	12
C_{rent}	0.1	% C_{land}	12
C_{ptax}	0.005	% FCI_j	12
C_{admin}	0.025	% TL_c	12
C_{ins}	0.2	% FCI_j	12

Abbreviations: C_{MT}, maintenance costs; CAPEX, capital expenditure; C_{sup}, supervision costs; L_c, operator labor cost; C_{os}, operating supply costs; FCI, fixed capital investment; C_{LC}, laboratory charges; $C_{\mathrm{P\&R}}$, patent and royalty costs; C_{OH}, overhead costs; C_{fin}, financing costs; C_{rent}, rent costs; C_{ptax}, local property tax; C_{admin}, administrative costs; C_{ins}, insurance costs; TL_c, total labor cost.

Supplementary Table 16: Miscellaneous raw materials costs.

Input Name	Value	Unit	Source
**AP SMR**	6,824,075.7	$ per year	2–4
**AP CCS**	12,434,783.5	$ per year	2–4
**AP BH_2S**	27,093,089.5	$ per year	2–4
**AP AEC**	335,267.1	$ per year	2–4

Abbreviations: AP, ammonia production; SMR, steam methane reforming; CCS, carbon capture and storage; BH_2S, blue hydrogen sulfide; AEC, alkaline electrolysis cell. Miscellaneous raw material costs are annual estimates based on data from Lewis et al., Brasington et al., and Young et al.[2–4].

Supplementary Table 17: Start-up costs.

Input Name	Value	Unit	Source
**AP SMR**	15,413,897.7	$	[2,4]
**AP CCS**	16,514,194.1	$	[2,4]
**AP BH$_2$S**	15,532,525.5	$	[2,4]
**AP AEC**	118,627.7	$	[2,4]

Abbreviations: AP, ammonia production; SMR, steam methane reforming; CCS, carbon capture and storage; BH_2S, blue hydrogen sulfide; AEC, alkaline electrolysis cell. Start-up costs are based on data from Lewis et al. and Brasington et al.[2,4].

Supplementary Table 18: GBM inputs.

Input Name	Value	Unit	Source
$P_{NG}(T=0)$	7.753	$ per mmBTU	[14]
μ_{NG}	-0.0016	-	[14]
σ_{NG}	0.039683	-	[14]
$P_{EL}(T=0)$	0.083	$ per kWh	[14]
μ_{EL}	-0.0006	-	[14]
σ_{EL}	0.00536	-	[14]

[A] The drift terms were obtained by calibrating the GBM model to the average price of commodity i in 2050 to the EIA's Annual Energy Outlook 2023 predictions[14]. Electricity was picked as the industrial electricity price in the baseline scenario. Natural gas was picked as the industrial natural gas price in the baseline scenario.

[A] The drift terms were obtained by calibrating the GBM model to the average price of commodity i in 2050 to the EIA's Annual Energy Outlook 2023 predictions[14]. Electricity was picked as the industrial electricity price in the baseline scenario. Natural gas was picked as the industrial natural gas price in the baseline scenario. Abbreviations: GBM, geometric Brownian motion; $P_{NG}(T=0)$, initial price of natural gas; μ_{NG}, drift term for natural gas; σ_{NG}, volatility of natural gas; $P_{EL}(T=0)$, initial price of electricity; μ_{EL}, drift term for electricity; σ_{EL}, volatility of electricity; mmBTU, million British thermal units; kWh, kilowatt-hour.

The OPEX is partitioned into two parts: a market-dependent OPEX ($MDOPEX_j$) and a market-independent (MI) OPEX ($MIOPEX_j$). These two values are combined to derive the $OPEX_j$ for each technology option considered.

The $OPEX_j$ is generally divided into fixed and variable costs. The fixed costs represent costs related to labor, overhead, maintenance, and insurance, while the variable costs rely on raw materials and utility costs. In the methodology we utilize, the fixed and variable costs are added together. From this combined quantity, we add another set of cost factors.

Some raw materials and utilities have costs that vary with the market; hence the variable costs are split between the $MDOPEX_j$ and $MIOPEX_j$. All the fixed costs are within the $MIOPEX_j$.

$$\text{OPEX} = \text{MIOPEX} + \text{MDOPEX} \tag{13}$$

The monthly cost of labor was calculated assuming a number of hours per day per processing step, H_{dp}. Then, dividing by the hours per shift, H_{shift}, and multiplying by the number of processing steps, P_{s}, times 7 days in a week over 5 shifts per week per operator, gives the number of operators assuming each operator can cover one shift per day. This is the first factor in the square brackets. The resulting hours an operator will work (5 shifts, 8 hours each) results in 40 hours per week. The hourly wage, wage_h, times 40 results in the weekly cost of an operator. This is the second factor in the square brackets. Finally, the weekly cost of labor (number of operators times the weekly cost per operator) is converted to the monthly cost of labor through the factor, $\frac{52}{12} \times F_{\text{a}}$, where F_{a} is the availability factor.

$$L_c = \left(\frac{H_{\text{dp}}}{H_{\text{shift}}} \times P_s \times \frac{7}{5} \right) \times (40 \times \text{wage}_h) \times \frac{52}{12} \times F_a \tag{14}$$

The total labor costs are a function of L_c, CAPEX_{j}, and cost factors. Hence, we introduce the total labor costs, TL_c:

$$\begin{aligned} \text{TL}_{c_j} = {} & L_c \left(1 + C_{\text{sup}} + C_{\text{LC}}\right) + \text{FCI}_j \times \frac{C_{\text{MT}}}{12} \\ & + \left(\text{FCI}_j \times \frac{C_{\text{MT}}}{12} \right) \times C_{\text{OS}} + \text{CAPEX}_j \times \frac{C_{\text{P\&R}}}{12} \\ & + \left(L_c \left(1 + C_{\text{sup}}\right) + \text{FCI}_j \times \frac{C_{\text{MT}}}{12} \right) \times C_{\text{OH}} \end{aligned} \tag{15}$$

There are also fixed charges, TF_c:

$$\text{TF}_{c_j} = \frac{C_{\text{fin}}}{12} \times \text{CAPEX}_j + \frac{C_{\text{rent}}}{12} \times C_{\text{land}} + \frac{C_{\text{ins}}}{12} \times \text{FCI}_j + \frac{C_{\text{ptax}}}{12} \times \text{FCI}_j + C_{\text{admin}} \times \text{TL}_c \tag{16}$$

After obtaining $TL_{c\text{j}}$ and $TF_{c\text{j}}$, we can calculate the fixed cost, FC:

$$\text{FC}_j = \text{TL}_{c_j} + \text{TF}_{c_j} \tag{17}$$

The utility costs portion of the MI OPEX_{j} is only the water costs since electricity is a market-dependent parameter. For SMR, CCS, and BH2S, supplementary equation (18) applies. We also add miscellaneous costs, which account for other raw materials (catalysts, column trays, solvents, water treatment, etc.). These miscellaneous costs are found in the original reports (see supplementary technical methods). We define the variable costs, VC_{j}, as:

$$\text{VC}_j = S_{\text{H}_2\text{O}} \times M_{\text{H}_2\text{O}_j} + \text{Misc}_j \tag{18}$$

If the process is AP AEC, the water dedicated to feedstock is water by reverse osmosis and is more expensive. Hence, for AP AEC, the following supplementary equation applies.

$$\text{VC}_{j=\text{AP AEC}} = S_{\text{H}_2\text{O}} \times M_{\text{H}_2\text{O}_j} + S_{\text{H}_2\text{O}_{\text{osmosis}}} \times M_{\text{H}_2\text{O}_{\text{osmosis}_j}} + \text{Misc}_j \tag{19}$$

With the previous definitions, the MI OPEX_{j} is defined as:

$$\text{MI OPEX}_j = \text{FC}_j + \text{VC}_j \tag{20}$$

For the market-dependent OPEX, the objective is to use geometric Brownian motion (GBM) to model natural gas, electricity, and ammonia prices with parameters including the drift, μ_i, and volatility, σ_i, for commodity i. We used a bivariate distribution for ammonia and natural gas prices so that we could set a correlation parameter between the two commodities. The correlation was calculated using industrial natural gas prices and ammonia price indices from December 2014 to January 2023[14].

$$P_i(T) = P(T=0)_i \prod_{T=0}^{L} \left[\mathcal{N}(1 + \mu_i, \sigma_i) \right] \tag{21}$$

Distribution and marketing costs (F_{DM}) and R&D (F_{RD}) costs are dependent on the variable costs. The $\mathrm{MDOPEX_j}$ can be defined as:

$$\begin{aligned}\mathrm{MDOPEX}_j(T) = M_{\mathrm{NG}} \times P_{\mathrm{NG}}(T) + M_{\mathrm{EL}} \times P_{\mathrm{EL}}(T) + \mathrm{MIOPEX}_j \\ + (F_{\mathrm{DM}} + F_{\mathrm{DM}} \times F_{\mathrm{RD}}) \times (M_{\mathrm{NG}} \times P_{\mathrm{NG}}(T) + M_{\mathrm{EL}} \times P_{\mathrm{EL}}(T) + \mathrm{MIOPEX}_j)\end{aligned} \tag{22}$$

where M_{NG} is the energy flowrate of natural gas in mmBTU per year, and M_{EL} is the electricity demand in kWh per year. The term, $M_{\mathrm{NG}} \times P_{\mathrm{NG}}(T) + M_{\mathrm{EL}} \times P_{\mathrm{EL}}(T) + MIOPEX_{\mathrm{z}}$, is the manufacturing cost. If it is the year when operations start, then start-up costs are added to the $\mathrm{MDOPEX_j(T)}$ (see supplementary table 16). With supplementary equation (22), supplementary equation (13) can be evaluated. More details on T and financial analysis in the economic methods section.

Economic Methods

This analysis employs a Stochastic Discounted Cash Flow (DCF) model to evaluate the economic viability of various technology pathways for ammonia production. This assessment aims to provide a comprehensive understanding of the economic performance by considering various uncertain factors and their impact on the Net Present Value (NPV) per lifetime ammonia produced metric. The NPV calculation factors in different cash flows, discount rates, and probabilities, enabling a robust evaluation of the pathways in a stochastic context.

The nomenclature for a Stochastic Discounted Cash Flow (DCF) model encompasses key financial and economic parameters used to evaluate investments and projects under uncertain conditions. These parameters define discount rates, costs, revenues, emissions, and various financial factors. These parameters are essential for assessing the potential financial outcomes of an investment while considering factors like inflation, tax credits, cash flows, and environmental impacts.

Economic Methods for Discounted Cash Flow model

The nomenclature for this this section is supplementary table 19.

Supplementary Table 19: Nomenclature for DCF section.

Parameter	Description	Units
d_p	Private discount rate	%
d_s	Social discount rate	%
e	Equity	-
R_e	Cost of equity	-
R_d	Cost of debt	-
ϕ_{state}	State income taxes	$
ϕ_{federal}	Federal income taxes	$
r	Interest rate	%
$\dot{M}_{\text{NH}_3}$	Monthly ammonia production	Tonne per day
S	Month of project start	month
C	Construction time	months
L	Operating lifetime (duration)	months
NPV	Net Present Value	$
$CF(T)$	Cash flow at time T	$
$Land(T)$	Costs of purchasing land at time T	$
C_{land}	Land cost	$
$WC(T)$	Costs of injecting working capital at time T	$
L_{loan}	Loan lifetime	years
H_y	Hours per year (365 * 24)	hour per year
$CI_{j_{\text{direct}}}$	Direct emission	$kgCO_2$ per kgH_2
$CI_{j_{\text{NG}}}$	Natural gas emission	$kgCO_2$ per kgH_2
$CI_{j_{\text{Biomass}}}$	Biomass emission	$kgCO_2$ per kgH_2
$X_{\text{oil}}(T)$	Electric demand from oil over the hydrogen production	$kgCO_2$ per kWh_e
$X_{\text{nuclear}}(T)$	Electric demand from nuclear over the hydrogen production	$kgCO_2$ per kWh_e
$X_{\text{renewables}}(T)$	Electric demand from renewables over the hydrogen production	$kgCO_2$ per kWh_e
$X_{\text{NG}}(T)$	Electric demand from NG over the hydrogen production	$kgCO_2$ per kWh_e
$X_{\text{coal}}(T)$	Electric demand from coal over the hydrogen production	$kgCO_2$ per kWh_e
$Sales(T)$	Revenue stream from selling ammonia at time T	$
$Credits_{\text{CE}}(T)$	Cash-equivalent tax credits	-
$F_{45\text{V}}(CI_j)$	Intensive tax credits per unit of H_2 captured	-
$F_{45\text{Q}}$	Intensive tax credits per unit of CO_2 captured	-
$L_{\text{equipment}}$	Equipment lifetime	years
$C_{\text{uninstalled equipment}}$	Costs of uninstalled equipment	$
η_{NGCC}	NGCC thermal efficiency	%

Abbreviations: DCF, discounted cash flow; d_p, private discount rate; d_s, social discount rate; R_e, cost of equity; R_d, cost of debt; ϕ_{state}, state income taxes; ϕ_{federal}, federal income taxes; r, interest rate; $\dot{M}_{\text{NH}_3}$, monthly ammonia production; NPV, net present value; $CF(T)$, cash flow at time T; $WC(T)$, working capital costs at time T; L_{loan}, loan lifetime; H_y, hours per year; CI, carbon intensity; $X_{\text{fuel}}(T)$, electric demand from a specific fuel source over hydrogen production; $F_{45\text{V}}$, tax credits for hydrogen; $F_{45\text{Q}}$, tax credits for CO_2 capture; $L_{\text{equipment}}$, equipment lifetime; η_{NGCC}, natural gas combined cycle efficiency; $kgCO_2$, kilograms of carbon dioxide; kWh_e, kilowatt-hour of electricity.

The economic performance of each technology pathway was assessed using a Net Present Value (NPV) per lifetime ammonia produced metric. NPV is the sum of the present value of all cash flows at each period (monthly basis) over the lifetime amount of ammonia production, $M_{\text{NH}\cdot 3}$.

$$\text{NPV} = \frac{1}{M_{\text{NH}_3}} \sum_{T=S}^{S+C+L} \frac{\text{CF}(T)}{(1+\frac{d_p}{12})^{(T-S)}} \tag{23}$$

where $CF(T)$ is the cash flow at time T. The public discount rate, d_p, was calculated as the weighted average cost of capital (WACC) (supplementary equation (23)); as shown below; e is the equity, R_e is the

cost of equity, R_d is the cost of debt, and $\phi_{\text{state}} + \phi_{\text{federal}}$ are the state and federal income taxes, respectively.

$$d_p = e \cdot R_e + ([1-e] \cdot R_d \cdot [1 - (\phi_{\text{state}} + \phi_{\text{federal}})]) \tag{24}$$

$$\begin{aligned} \text{CF}(T) &= \text{FCI}(T) + \text{Land}(T) + \text{WC}(T) + \text{PMT}(T) \\ &\quad + \text{Sales}(T) + \text{OPEX}(T) + \text{Tax}(T) + \text{Credits}_{\text{CE}}(T) \end{aligned} \tag{25}$$

Supplementary equations (26–28) represent the staggered spending of the FCI to build the ammonia plant over three years (C months). Y_1, Y_2, and Y_3 are the fractions of the FCI that are spent in a given year.

$$FCI(0 < T \leq \frac{C}{3}) = -Y_1 \cdot \frac{FCI_j}{12} \tag{26}$$

$$FCI\left(\frac{C}{3} < T \leq \frac{2C}{3}\right) = -Y_2 \cdot \frac{FCI_j}{12} \tag{27}$$

$$FCI\left(\frac{2C}{3} < T \leq C\right) = -Y_3 \cdot \frac{FCI_j}{12} \tag{28}$$

Land(T) and WC(T) are the costs of purchasing land and injecting working capital to begin operation.

$$\text{Land}(T = S) = -C_{\text{land}}, \quad \text{Land}(T = S + C + L) = C_{\text{land}} \tag{29}$$

$$\text{WC}(T = S + C) = -\text{WC}_j, \quad \text{WC}(T = S + C + L) = \text{WC}_j \tag{30}$$

Supplementary equations (31)-(33) represent increasing interest payments as the amount of borrowed capital increases throughout the construction period and 34 the constant payments to pay off the loan.

$$\text{PMT}(S < T \leq S + \frac{C}{3}) = -\frac{r}{12} \times T \times (1-e) \times Y_1 \times \frac{\text{FCI}_j}{12} \tag{31}$$

$$\text{PMT}(S + \frac{C}{3} < T \leq S + \frac{2C}{3}) = -\frac{r}{12} \times (1-e) \times \left[\left(T - \left(S + \frac{C}{3}\right)\right) \times \frac{Y_2 \times \text{FCI}_j}{12} + Y_1 \times \text{FCI}_j\right] \tag{32}$$

$$\text{PMT}(S + \frac{2C}{3} < T \leq S + C) = -\frac{r}{12} \times (1-e) \times \left[\left(T - \left(\frac{2C}{3} + S\right)\right) \times \frac{Y_3 \times \text{FCI}_j}{12} + (Y_1 + Y_2) \times \text{FCI}_j\right] \tag{33}$$

$$\text{PMT}(S + C < T \leq S + C + L_{\text{loan}}) = -\text{FCI}_j \times \frac{r}{1 - (1-r)^{12 \times L_{\text{loan}}}} \tag{34}$$

Sales(T) and OPEX(T) represent the revenue stream from selling ammonia to the market and the cost of operating the plant, respectively.

$$\text{Sales}(S + C < T \leq S + C + L) = \dot{M}_{\text{NH}_3} \times \left(\frac{F_A \times H_y}{24}\right) \times \frac{1}{12} \times P_{\text{NH}_3}(T) \tag{35}$$

$$\text{OPEX}(S + C < T \leq S + C + L) = \text{OPEX}_j(T) \tag{36}$$

where L_{loan} is the loan lifetime in years, $F_a \times H_y$ are the operating hours per year, $\dot{M}_{\text{NH}\cdot 3}$ is the monthly ammonia production, and $C_{\text{NH}\cdot 3}(T)$ is its market-dependent cost.

Tax(T) is the income tax, and Credits$_{\text{CE}}(T)$ represents the cash-equivalent IRA tax credits. The following variables are interconnected in the financial analysis of income and expenses, with depreciation and uninstalled equipment cost playing key roles in determining net revenue and, subsequently, tax.

$$\text{Tax}(S + C < T < S + C + L) = -\text{Net revenue}(T) \times (\phi_{\text{state}} + \phi_{\text{federal}}) \tag{37}$$

$$\begin{aligned}\text{Net revenue}(S+C \leq T \leq S+C+L) = &\text{Depreciation}(S+C \leq T \leq S+C+L_{\text{equipment}}) \\ &+ \text{OPEX}(T) \\ &+ \text{Sales}(T) \\ &+ \text{PMT}(T)\end{aligned} \tag{38}$$

$$\text{Depreciation}(T) = \left(\frac{100\%}{L_{\text{equipment}}}\right) \times \text{EC}_{u_j} \tag{39}$$

Economic Methods for Policy model

The calculation of the carbon intensity as a function of time, $CI_{\text{j}}(T)$, is divided into multiple components. Each component is in units of $\frac{\text{Kg}_{\text{CO}_2}}{\text{Kg}_{\text{H}_2}}$ in supplementary equation (40). The electric grid carbon intensity (C.19) in $\frac{\text{Kg}_{\text{CO}_2}}{\text{Kg}_{\text{H}_2}}$ is the electric mix weighted average of the carbon intensities of each type of electric generation times the electric demand of the technology j over the hydrogen production. In this study, we use the electricity demand of hydrogen only because the tax credits only depend on the emissions of hydrogen.

$$\text{CI}_j(T) = \text{CI}_{j_{\text{direct}}} + \text{CI}_{j_{\text{NG}}} + \text{CI}_{j_{\text{Biomass}}} + \text{CI}_{j_{\text{Electricity}}}(T) \tag{40}$$

$$\begin{aligned}\text{CI}_{j_{\text{Electricity}}}(T) = &X_{\text{oil}}(T) \times \text{CI}_{\text{elec\_oil}} + X_{\text{nuclear}}(T) \times \text{CI}_{\text{elec\_nuclear}} \\ &+ X_{\text{renewables}}(T) \times \text{CI}_{\text{elec\_nuclear}} + X_{\text{NG}}(T) \times \text{CI}_{\text{elec\_NG}} \\ &+ X_{\text{coal}}(T) \times \text{CI}_{\text{elec\_coal}}\end{aligned} \tag{41}$$

The natural gas carbon intensity of SMR and CCS are estimated from the upstream emissions per kWh of electricity of an NGCC plant times the average efficiency of an NGCC plant (see supplementary table 20). The direct, or stack, emissions, $CI_{\text{j\_direct}}$, from SMR and CCS come from Lewis et al.[2] and are assumed to be zero for BH2S and AEC.

Supplementary Table 20: Direct carbon emissions intensity.

Input Name	Value	Unit	Source
$CI_{\text{SMR}}^{\text{direct}}$	9.3	Kg CO_2 per Kg H_2	2
η_{NGCC}	46.1%	-	15

Efficiency value for NGCC. Abbreviations: $CI_{\text{SMR}}^{\text{direct}}$, direct carbon intensity of steam methane reforming; η_{NGCC}, natural gas combined cycle efficiency; NGCC, natural gas combined cycle; Kg CO_2, kilograms of carbon dioxide; Kg H_2, kilograms of hydrogen.

$$\text{CI}_{\text{SMR}_{\text{NG}}} = \text{CI}_{\text{elec\_NG}} \times \eta_{\text{NGCC}} \times \frac{\dot{M}_{\text{NG}_j}/365}{M_{\text{H}_2} \times h_{\text{NG}}^{\text{LHV}} \times [0.000948 \text{ MMBtu/MJ}] \times [1000 \text{ kg/tonne}]} \tag{42}$$

$$\text{CI}_{\text{CCS}_{\text{NG}}} = \text{CI}_{\text{SMR}_{\text{NG}}} \times (1 - R_{\text{CO}_2}) \tag{43}$$

$$\text{CI}_{j_{\text{Biomass}}} = \frac{\dot{M}_{\text{biomass}}}{M_{\text{H}_2}} \times \text{CI}_{\text{Biomass}} \tag{44}$$

The AP plan first must decide on a credit program (45V or 45Q). AP BH2S and AP AEC do not qualify for 45Q. Hence, only AP CCS can choose between 45V and 45Q by using the formula below:

$$\max\left[45\text{V}: \sum_{T=S+C}^{L+S+C} \mathbf{H}_{\text{CE}} \left(\frac{F_{45Q} \times \dot{M}_{\text{H}_2} \times (\text{CI}_{\text{SMR}_{\text{direct}}} - \text{CI}_{j_{\text{direct}}})}{(1+d_p)^{T-S}} \right), \right. \\ \left. 45\text{Q}: \sum_{T=S+C}^{L+S+C} \mathbf{H}_{\text{CE}} \left(\frac{\dot{M}_{\text{H}_2} \times F_{45V} \times \text{CI}_j}{(1+d_p)^{T-S}} \right) \right] \tag{45}$$

where $\mathbf{H}_{\text{CE}}$ is an operator that converts the tax credits from the IRA-eligible tax credits to the cash-equivalent utilizing the "direct pay" and "transferability" capabilities of the credits – it performs the logical operation in supplementary equations (48) and (49). The first term in the max function is the NPV of 45V credits. The second term is the NPV of 45Q credits.

$$\text{Credits}_{45V_j}(T) = \dot{M}_{\text{H}_2} \times F_{45V} \times \text{CI}_j \tag{46}$$

$$\text{Credits}_{45Q_j}(T) = F_{45Q} \times \dot{M}_{\text{H}_2} \times (\text{CI}_{\text{SMR}_{\text{direct}}} - \text{CI}_{j_{\text{direct}}}) \tag{47}$$

$$\text{Credits}_{\text{CE}}(T \mid\mid \text{Tax}(T) \mid> \text{Credits}(T)) = \text{Credits}_j(T) \tag{48}$$

$$\text{Credits}_{\text{CE}}(T \mid\mid \text{Tax}(T) \mid< \text{Credits}(T)) = -\text{Tax}(T) + F_t(T) \times (\text{Credits}_j(T) + \text{Tax}(T)) \tag{49}$$

where $F_{45\text{V}}(CI_{\text{j}})$ and $F_{45\text{Q}}$ are the intensive tax credits per unit of H_2 produced and CO_2 captured, respectively. 45V is a piecewise function of CI. 45V and 45Q are mutually exclusive, so the highest tax credit is preferred for technology option j. $\dot{M}_{\text{H}\cdot 2}$ is the monthly flowrate of H_2 and CO_2. $F_t(T)$ is an exchange rate of USD per tax credit. It is equal to 1 in the first five years of operation (due to direct pay) and then attains a market value of less than one after five years. $F_{45\text{V}}(CI_{\text{j}})$ and $F_{45\text{Q}}$ become zero after 10 and 12 years of operation, respectively.

Economic Methods for Measurement metrics

A valuable measure in the context of comparisons of climate policy instruments could be the carbon abatement cost (CAC_{j}), which quantifies the cost to the taxpayer of bringing low-carbon technologies to commercialization, normalized by the mitigated emissions over the lifetime of the plant.

$$\text{CAC}_j = \frac{\sum_{T=S+C}^{L+S+C} \left(\frac{\text{Credits}_j(T)}{[1+d_s]^{T-S}} \right)}{\sum_{T=S+C}^{L+S+C} \left(\frac{\dot{M}_{\text{H}_2} \times (\text{CI}_{\text{SMR}}(T) - \text{CI}_j(T))}{[1+d_s]^{T-S}} \right)} \tag{50}$$

where $L + S + C$ represents the operating lifetime, the month the plant begins to be built, and the construction period, respectively. The denominator represents the total abated emissions as a Riemann sum of the CO_2 at all periods. Both the carbon and credits are discounted to the present value at the social discount rate, d_s. The discount rate for the CAC is set to two percent, in line with EPA's estimates of the social cost of carbon[16].

Much like the CAC_j, the total tax credits we measure are on a hydrogen basis. This metric is easily compared to the popular levelized cost of hydrogen (LCOH) metric. We develop the following metrics to quantify the three types of tax credit $\text{TC}_{j\alpha}$ quantifications we report:

$$\text{TC}_{j_{\text{Potential (2\%)}}} = \frac{\sum_{T=S+C}^{L+S+C} \left(\frac{\text{Credits}_j(T)}{[1+d_s/12]^{T-S}} \right)}{\sum_{T=S+C}^{S+C+L_{\text{H}_2}} \left(\frac{\dot{M}_{\text{H}_2}}{[1+d_s/12]^{T-S}} \right)} \tag{51}$$

$$\text{TC}_{j_{\text{CE (2\%)}}} = \frac{\sum_{T=S+C}^{L+S+C} \left(\frac{\text{Credits}_{\text{CE}_j}(T)}{[1+d_s/12]^{T-S}} \right)}{\sum_{T=S+C}^{S+C+L_{\text{H}_2}} \left(\frac{\dot{M}_{\text{H}_2}}{[1+d_s/12]^{T-S}} \right)} \tag{52}$$

$$\mathrm{TC}_{j_{\mathrm{CE}\ (9.3\%)}} = \frac{\sum_{T=S+C}^{L+S+C}\left(\frac{\mathrm{Credits}_{\mathrm{CE}_j}(T)}{[1+d_p/12]^{T-S}}\right)}{\sum_{T=S+C}^{S+C+L_{\mathrm{H}_2}}\left(\frac{\dot{M}_{\mathrm{H}_2}}{[1+d_p/12]^{T-S}}\right)} \tag{53}$$

We take the NPV of the tax credits from the public and private perspectives. $TC_{\mathrm{j\_Potential}\ (2\%)}$ captures the total amount of credits issued – hence the cost to the taxpayer. $TC_{\mathrm{j\_CE}\ (2\%)}$ is always less than $TC_{\mathrm{j\_Potential}\ (2\%)}$ and represents the amount of tax credits awarded to the low-carbon AP investor from the total pool of tax credits, $TC_{\mathrm{j\_Potential}\ (2\%)}$. $TC_{\mathrm{j\_CE}\ (9.3\%)}$ is the low-carbon AP investor's valuation of the tax credits using the WACC as the discount rate. The AP investor is more "impatient" than the government and hence places more value on credits awarded in the near future.

Supplementary Table 21: IRA tax credit market value assumptions for "direct pay" and "transferability" provisions.

**Time**	**Value**	**Notes**	**Source**
Years 0-5 of operation	1 $ per TC	Per IRS "direct pay" guidelines published in June.	[17,18]
2031-2032	Uniform (0.6,0.85)	Early industry prediction	[19,20]
2032-2033	Uniform (0.7,0.9)	Early industry prediction	[19,20]
2033-2034	Uniform (0.75,0.925)	Early industry prediction	[19,20]
2034-2035	Uniform (0.8,0.95)	Early industry prediction	[19,20]
2035-onwards	Uniform (0.85,0.95)	Early industry prediction	[19,20]

Inflation Reduction Act's (IRA) tax credit market value assumptions for "direct pay" and "transferability" provisions. Abbreviations: IRS, Internal Revenue Service; TC, tax credit. Assumptions are based on early industry predictions and guidelines from referenced sources.

Supplementary Table 22: Nomenclature.

**Parameter**	**Description**	**Units**
CAC_j	Carbon abatement cost for technology j	\$ per tCO_2eq
d_s	Social discount rate	%
TC_{j_α}	Tax credit for technology j for type of credit α	\$ per Kg H_2
α	Can be the potential tax credits discounted at d_s, cash-equivalent tax credits discounted at d_s, or cash-equivalent tax credits discounted at d_p	
L_{H_2}	Lifetime of hydrogen produced under the IRA	months

Abbreviations: CAC_j, carbon abatement cost for technology j; d_s, social discount rate; TC_{j_α}, tax credit for technology j for type of credit α; L_{H_2}, lifetime of hydrogen produced under the Inflation Reduction Act (IRA); tCO_2eq, tonnes of CO_2 equivalent; Kg H_2, kilograms of hydrogen.

Supplementary Table 23: Carbon intensity inputs for electricity mix.

Input Name	Value	Unit	Source
$X_{\text{oil}}(T)$	uniform(0.256, 1.17)	Kg CO_2 per Kg H_2	21
$X_{\text{NG}}(T)$	uniform(0.389, 0.988)	Kg CO_2 per Kg H_2	21
$X_{\text{Coal}}(T)$	uniform(1.001, 1.01)	Kg CO_2 per Kg H_2	21
$X_{\text{nuclear}}(T)$	uniform(0.012, 0.220)	Kg CO_2 per Kg H_2	21
$X_{\text{renewables}}(T)$	0	Kg CO_2 per Kg H_2	21

Abbreviations: $X_{\text{oil}}(T)$, carbon intensity from oil in the electricity mix; $X_{\text{NG}}(T)$, carbon intensity from natural gas in the electricity mix; $X_{\text{Coal}}(T)$, carbon intensity from coal in the electricity mix; $X_{\text{nuclear}}(T)$, carbon intensity from nuclear in the electricity mix; $X_{\text{renewables}}(T)$, carbon intensity from renewables in the electricity mix; Kg CO_2, kilograms of carbon dioxide; Kg H_2, kilograms of hydrogen. Values represent uniform distributions based on data from Nicholson (2021)[21].

Supplementary Table 24: Financial inputs.

Input Name	Value	Unit	Source
F_a	0.9	-	2
Y_1, Y_2, Y_3	10%, 60%, 30%	-	Assumption
R_e	12.27%	-	22
R_d	5.5%	-	22
e	63.19%	-	22
ϕ_{state}	5.25%	-	
ϕ_{federal}	21%	-	
L_{loan}	180	years	2
$L_{\text{equipment}}$	84	years	10

Abbreviations: F_a, availability factor; Y_1, Y_2, Y_3, distribution of costs over the first three years; R_e, cost of equity; R_d, cost of debt; e, equity fraction; ϕ_{state}, state income tax rate; ϕ_{federal}, federal income tax rate; L_{loan}, loan lifetime; $L_{\text{equipment}}$, equipment lifetime.

Supplementary Table 25: IRA Inputs.

Input Name	Value	Unit	Source
F_{45V} (0 – 0.45 $KgCO_{2eq}$ per Kg H_2)	3	\$ per Kg H_2	[23]
F_{45V} (0.45 – 1.5 $KgCO_{2eq}$ per Kg H_2)	1	\$ per Kg H_2	[23]
F_{45V} (1.5 – 2.5 $KgCO_{2eq}$ per Kg H_2)	0.75	\$ per Kg H_2	[23]
F_{45V} (2.5 – 4.0 $KgCO_{2eq}$ per Kg H_2)	0.6	\$ per Kg H_2	[23]
F_{45Q}	85	\$ per tCO_2e	[23]

Abbreviations: F_{45V}, tax credit for hydrogen production based on carbon intensity; F_{45Q}, tax credit for CO_2 sequestration; $KgCO_{2eq}$, kilograms of CO_2 equivalent; Kg H_2, kilograms of hydrogen; tCO_2e, tonnes of CO_2 equivalent. Tax credits are based on thresholds defined in the Inflation Reduction Act (IRA) [23].

Supplementary Table 26: Measurement metrics values.

Input Name	Value	Unit	Source
d_s	2%	% per year	[16]
L_{H_2}	120^A	months	[23]

^{A} The lifetime of 45V credits (10 years) is assumed to be the lifetime of hydrogen production under the IRA. Abbreviations: d_s, social discount rate; L_{H_2}, lifetime of hydrogen production under the IRA.

Electricity Source Methods

The problem aims to minimize the capital cost required for installations to meet constant electricity demand. Optimal sizing of solar, wind and battery capacity depends on the relative costs, resource availability, and requirements for constant electricity output. Thus, the objective is to find the optimal capacities of asset installations that minimize the overall capital cost.

Supplementary Table 27: Nomenclature.

Description	Units	Source
**Decision variables**		
w	Installed wind capacity	MW
b	Installed battery capacity	MW
S	Installed solar capacity	MW
$w(t)$	Wind generation in each time period	MWh
$s(t)$	Solar generation in each time period	MWh
$c(t)$	Battery charge in each time period	MWh
$d(t)$	Battery discharge in each time period	MWh
$b(t)$	Battery state of charge in each time period	MWh
**Parameters**		
$CF_w(t)$	Capacity factor of wind across different periods	Unitless
$CF_s(t)$	Capacity factor of solar across different periods	Unitless
D	Constant electricity demand, varies by specific technologies	MWh
EFF	Roundtrip efficiency of the battery system, assumed 85%[A]	Unitless
$COST_{\text{wind}}$	Capital cost per MW of wind capacity	\$ per MW
$COST_{\text{battery}}$	Capital cost per MW of battery capacity	\$ per MW
$COST_{\text{solar}}$	Capital cost per MW of solar capacity	\$ per MW

[A] Cole & Frazier (2019). Abbreviations: MW, megawatt; MWh, megawatt-hour; $CF_w(t)$, capacity factor of wind across different periods; $CF_s(t)$, capacity factor of solar across different periods; EFF, roundtrip efficiency of the battery system; $COST_{\text{wind}}$, capital cost per MW of wind capacity; $COST_{\text{battery}}$, capital cost per MW of battery capacity; $COST_{\text{solar}}$, capital cost per MW of solar capacity.

Electricity Source Methods for Formulation

The objective function (supplementary equation (54)) is to minimize the total capital investments, which is the sum of the capital cost of wind installation ($\text{COST}_{\text{wind}} \times w$), solar ($\text{COST}_{\text{solar}} \times s$), and the capital cost of battery installation ($\text{COST}_{\text{battery}} \times b$).

$$\min_{\substack{w\geq 0,\\ b\geq 0,\\ s\geq 0}} \quad F = \text{COST}_{\text{wind}} \times w + \text{COST}_{\text{battery}} \times b + \text{COST}_{\text{solar}} \times s \tag{54}$$

The power balance constraint (55) ensures that the net power balance in each time period equals the electricity demand (D). It accounts for wind generation $w(t)$, battery charging $c(t)$, solar generation $s(t)$, and battery discharging $d(t)$. The net power balance is achieved by subtracting the battery charge and adding the battery discharge to the wind generation:

$$s(t) + w(t) - c(t) + d(t) = D, \ \forall t \tag{55}$$

Wind and solar generation constraints (56) and (57) enforce generation in each time period to not exceed resource availability $\text{CF}(t) \times w$:

$$0 \leq w(t) \leq \text{CF}_{\text{wind}}(t) \times w, \ \forall t \tag{56}$$

$$0 \leq s(t) \leq \text{CF}_{\text{solar}}(t) \times s, \ \forall t \tag{57}$$

Supplementary equations (58) and (59) limit the battery charging and discharging to their capacity:

$$0 \leq c(t) \leq b, \ \forall t \tag{58}$$

$$0 \leq d(t) \leq b, \ \forall t \tag{59}$$

Supplementary equation (60) defines the battery storage state considering roundtrip efficiency, charge, and discharge dynamics:

$$b(t) = b(t-1) + \text{EFF} \times c(t) - d(t), \ \forall t > 1 \tag{60}$$

Lastly, supplementary equation (61) puts a lower and an upper limit on the battery's state of charge:

$$0 \leq b(t) \leq b, \ \forall t \tag{61}$$

The data for the capacity factors $CF(t)$ is parameterized by location, year, and design. The hourly capacity data originates from Pfenninger et al.[24].

Locations: The capacity factors are obtained from 8 locations, labeled Yara, Koch, Woodward, Port Neal, Verdigris, Nutrien, Donaldson, and AdvanSix. The locations match some of the largest AP plants in the US (see supplementary table 28).

Supplementary Table 28: Specific locations of AP plants.

**Company**	**Name**	**State**	**Latitude**	**Longitude**
CF Industries	Donaldsonville Complex	LS	30.087397	-90.955682
CF Industries	Verdigris Complex	OK	36.233335	-95.718833
CF Industries	Woodward Complex	OK	36.437942	-99.472056
CF Industries	Port Neal Complex	IA	42.332879	-96.377213
Koch Industries	Koch Fertilizer Company Enid	OK	36.380941	-97.761921
Nutrien	Nutrien Augusta Nitrogen	GA	33.443125	-81.930376
Yara	BASF Chemicals Division	TX	29.000639	-95.393318
AdvanSix	AdvanSix	VA	37.300405	-77.271941

Abbreviations: AP, ammonia production; LS, Louisiana; OK, Oklahoma; IA, Iowa; GA, Georgia; TX, Texas; VA, Virginia. Latitude and longitude coordinates represent the geographical locations of specific ammonia production plants.

Supplementary Table 29: Constant electricity demand of each technology.

	**Low**		**High**	
	**MW**	**MWh per year**	**MW**	**MWh per year**
AP CCS, D	117	922,428		
AP BH2S, D	127	1,001,268		
AP AEC, D	913	7,198,092	1,007	7,939,188

Abbreviations: AP, ammonia production; CCS, carbon capture and storage; BH_2S, blue hydrogen sulfide; AEC, alkaline electrolysis cell; MW, megawatt; MWh per year, megawatt-hours per year. Notes: AP AEC has a demand range because we assume an uncertain electrolyzer efficiency.

Year: Only 2019 data was available. We assume 2019 capacity factors to be a typical meteorological year (TMY) for the AP plant model for computational tractability. In other words, the time-varying results from this optimization problem $w(t), d(t)$, etc. are oscillatory functions in the context of the AP plant (see SI F).

Wind turbine design: As wind turbine technology improves, the costs are expected to decrease and the hub heights and capacity per turbine to increase. 2026 scenario data was obtained using a hub height of 90.2 m and a BONUS B82 2300 turbine. 2033 data was obtained using a hub height of 120 m and a GAMESA G128 5000 turbine. The turbine design was picked to match closely with the average design specifications

of deployed wind turbines in the baseline and moderate scenarios of NREL's Annual Technology Baseline report[25].

Our stochastic NPV model uses a uniform distribution to capture the lower and upper bounds of uncertain variables such as wind and battery CAPEX. To capture the upper and lower bounds of wind resource availability in those eight locations, we compute the average monthly and hourly capacity factors and pick two farm locations each year with the highest and lowest average capacity factors (see supplementary table 30). Intuitively, locations with low-energy wind resources will have a larger wind capacity (higher overall CAPEX) than locations with high-energy wind resources (lower overall CAPEX).

Supplementary Table 30: Capacity factor data for selected locations.

	2023			
**Location**	**Average**	**Minimum**	**Maximum**	**Std Deviation**
Koch	35.5%	0.0%	98.2%	28.1%
AdvanSix	17.8%	0.0%	97.6%	18.9%
Port Neal	34.9%	0.0%	98.2%	27.8%
Nutrien	18.4%	0.0%	98.2%	18.7%
Yara	29.1%	0.0%	98.1%	23.7%
Woodward	39.2%	0.0%	98.2%	29.6%
Donaldson	19.4%	0.0%	98.2%	20.1%
Verdigris	31.4%	0.0%	98.2%	26.7%
	**2030**			
**Location**	**Average**	**Minimum**	**Maximum**	**Std Deviation**
Koch	42.7%	0.0%	96.4%	29.8%
AdvanSix	26.7%	0.0%	96.4%	23.5%
Port Neal	41.9%	0.0%	96.4%	29.3%
Nutrien	25.4%	0.0%	96.4%	22.5%
Yara	35.7%	0.0%	96.4%	26.0%
Woodward	46.1%	0.0%	96.4%	30.9%
Donaldson	25.8%	0.0%	96.4%	23.2%
Verdigris	38.7%	0.0%	96.4%	28.8%

Abbreviations: Std Deviation, standard deviation. Capacity factor data represents the operational efficiency of the facilities at selected locations for the years 2023 and 2030. Values include average, minimum, maximum, and standard deviation of capacity factors for each location.

This helps in understanding which locations are performing the best and the worst in wind electricity generation, and these two locations' data are used for stochastic NPV analysis.

The capital cost of wind and battery is based on values from Bistline et al.[26]. See below in supplementary table 31.

Supplementary Table 31: CAPEX of wind and battery systems.

**Battery**	**Low**	**High**	**Units**
2023, $COST_{\text{battery}}$	800	1,500	$ per kW
2030, $COST_{\text{battery}}$	450	1,200	$ per kW
**Wind**	**Low**	**High**	**Units**
2023, $COST_{\text{wind}}$	1,200	1,400	$ per kW
2030, $COST_{\text{wind}}$	750	1,200	$ per kW

Abbreviations: CAPEX, capital expenditure; $COST_{\text{battery}}$, cost of battery systems; $COST_{\text{wind}}$, cost of wind systems; kW, kilowatt. The table presents the range of CAPEX values for wind and battery systems for the years 2023 and 2030.

Electricity Source Methods for Implementation

The optimization problem is formulated in Python and solved using the PuLP library, a popular open-source linear programming (LP) modeling package that can seamlessly handle mixed-integer linear programming (MILP) scenarios.

PuLP offers a simple and intuitive syntax for defining optimization problems using Python, such as intuitive syntax for defining decision variables, objective functions, and constraints. Thus, the model can be formulated to closely resemble the mathematical notation that describes optimization problems. It provides a high-level abstraction that makes it easier to express mathematical programming concepts.

Electricity Source Methods for Results

The optimal results are computed for installed wind capacity, battery capacity, wind generation, battery charging, battery discharging, battery state of charge, storage duration, and wind supply curtailment. These results collectively provide insights into how the optimization model has determined the optimal configuration for wind capacity, battery capacity, and their operational behavior to minimize costs while ensuring supply-demand balance and considering various constraints. We report and publish all results from this optimization in an Excel file named *AP_NE_Optimization_Results.xlsx.*

Scenario-Specific Methods

Supplementary Table 32: Business models and policy scenarios.

**Scenario**	**Description**	**AP SMR**	**LCAP**
A	"IRA compatible" US power grid (with a range of LCA of fuels): Grid electricity (2023-2035) – EIA AEO 2023 scenario (CO2 intensity)	Yes	Yes
B	"Build and own" a wind farm with a battery that powers the AP processes (upfront CAPEX-intensive, benefit of low marginal cost of electricity generation)[a]	No	Yes
C	Power Purchase Agreement (PPA) with a wind farm with a battery (OPEX-intensive, electricity purchase paid at the LCOE of wind farm with battery)[a]	No	Yes

[a] We developed an optimization model to minimize the CAPEX of the wind and battery system. The main constraint of this system is constant electric output. For more details, see the supplementary electricity source methods section. Abbreviations: AP, ammonia production; SMR, steam methane reforming; LCAP, low-carbon ammonia production; CAPEX, capital expenditure; OPEX, operational expenditure; LCOE, levelized cost of electricity; IRA, Inflation Reduction Act; EIA AEO, Energy Information Administration Annual Energy Outlook.

Supplementary Table 33: Nomenclature for scenario-specific changes

Variables/Parameters	Description	Units
$MIOPEX_{\mathrm{el}}(\Gamma)$	Additional market-independent OPEX as a function of the remainder of the AP plant time attributed to the solar, wind and battery O&M.	\$ per month
$d_{\mathrm{monthly}}(\Gamma)$	Monthly aggregated sum of the battery discharge	MWh per month
$C^{\mathrm{wind}}_{(f+v)}$	Fixed and variable O&M cost of the wind farm	\$ per MW per year
$C^{\mathrm{solar}}_{(f+v)}$	Fixed and variable O&M cost of the solar farm	\$ per MW per year
C^{battery}_{f}	Fixed O&M costs of the battery system	\$ per kW-year
C^{battery}_{v}	Variable O&M costs of the battery system	\$ per MWh
L	Plant operating lifetime	months
S	The time step in which the plant construction begins	month
C	Construction time	months
$S_{\mathrm{el}}(T)$	Surplus electricity income	\$ per month
$w_{\mathrm{monthly}}(\Gamma)$	Monthly aggregated sum of the total electricity generated by the wind farm	MWh per month
$s_{\mathrm{monthly}}(\Gamma)$	Monthly aggregated sum of the total electricity generated by the solar farm	MWh per month
$LCOE_{\mathrm{wind\ farm\ only}}$	The levelized cost of electricity of a stand-alone wind and solar farm	\$ per MWh
$P_{\mathrm{Electricity}}(T)$	Electricity market price	\$ per kWh
$48E$	The percentage ITC of 48E credits from the IRA	% solar, wind and battery CAPEX
$45Y(T)$	Time function of 45Y credits bounded by the start of operations and 45Y credit lifetime, E_{45Y}	\$ per MWh
E_{45Y}	Lifetime of 45Y credits, constrained by $2023 + \frac{(E_{45Y}+S+C)}{12} = 2050$	Months
$H_{\mathrm{CE}}[x]$	Cash-equivalent operator, performs the conversion from nominal tax credits to cash-equivalent tax credits	\$
d_r	Discount rate	
$LCOE$	Levelized cost of electricity	\$ per MWh
CO_2 tax	CO_2 tax imposed by CBAM	\$ per Month
$CI_{\mathrm{SMR_{EU}}}(T)$	European AP SMR carbon intensity	Kg CO_2eq per kg H_2
$M_{\mathrm{H_2}}$	Mass flowrate of hydrogen	Tonnes per day
F_a	Availability factor	%
CO_2^{EUPrice}	Price for CBAM certificates	\$ per tCO_2
$R_{\mathrm{EU\ emissions}}$	Decay rate for EU AP SMR emissions intensity	%
L_{battery}	Lifetime of battery system	years
L_{wind}	Lifetime of wind farm turbines	years
L_{solar}	Lifetime of solar farm turbines	years

Abbreviations: $MIOPEX_{\mathrm{el}}(\Gamma)$, additional market-independent OPEX for electricity systems; $d_{\mathrm{monthly}}(\Gamma)$, monthly battery discharge; $C^{\mathrm{wind}}_{(f+v)}$, fixed and variable O&M costs of wind farms; $C^{\mathrm{solar}}_{(f+v)}$, fixed and variable O&M costs of solar farms; C^{battery}_{f}, fixed O&M costs of battery systems; C^{battery}_{v}, variable O&M costs of battery systems; L, plant operating lifetime; S, construction start time; C, construction time; $S_{\mathrm{el}}(T)$, surplus electricity income; $w_{\mathrm{monthly}}(\Gamma)$, monthly wind generation; $s_{\mathrm{monthly}}(\Gamma)$, monthly solar generation; $LCOE_{\mathrm{wind\ farm\ only}}$, levelized cost of electricity for stand-alone wind and solar farms; $P_{\mathrm{Electricity}}(T)$, electricity market price; $48E$, ITC percentage from IRA credits; $45Y(T)$, 45Y credits function; E_{45Y}, lifetime of 45Y credits; $H_{\mathrm{CE}}[x]$, cash-equivalent operator; d_r, discount rate; $LCOE$, levelized cost of electricity; CO_2 tax, CO_2 tax from CBAM; $CI_{\mathrm{SMR_{EU}}}(T)$, EU SMR carbon intensity; $M_{\mathrm{H_2}}$, hydrogen flowrate; F_a, availability factor; CO_2^{EUPrice}, CBAM certificate price; $R_{\mathrm{EU\ emissions}}$, EU SMR emission decay rate; L_{battery}, battery system lifetime; L_{wind}, wind farm turbine lifetime; L_{solar}, solar farm turbine lifetime; Kg CO_2eq, kilograms of CO_2 equivalent; tCO_2, tonnes of CO_2; MWh, megawatt-hour; kWh, kilowatt-hour; TPD, tonnes per day; CBAM, Carbon Border Adjustment Mechanism.

Supplementary Table 34: Input values for for scenario-specific section.

**Variables/Parameters**	**Value**	**Source**
$C^{\text{wind}}_{(f+v)}$	52.22 $ per MW per year	27
$C^{\text{solar}}_{(f+v)}$		
C^{battery}_{f}	Uniform(6.16, 49.33) $ per kW-year	28
C^{battery}_{v}	Uniform(0, 8.63) $ per MWh	28
L	480 months (40 years)	Author assumption
S	0 months (year 2023) or 84 (year 2030)	Author assumption
C	36 months	Author assumption
$48E$	Uniform(30%, 40%)	23
$45Y(T)$	$1.5 per MWh (when eligible)	23
M_{H_2}	483 TPD	2
F_a	90%	2
L_{battery}	Uniform(13, 20) years	28
L_{wind}	20 years	27
L_{solar}	20 years	
$CI_{\text{SMR}_{\text{EU}}}(T=0)$	8.82 ($KgCO_2e$) per (KgH_2)	29
$R_{\text{EU emissions}}$	1.4%	29–31
CO_2^{EUPrice}	Uniform(35-100) $ per tCO_2e	29

Abbreviations: $C^{\text{wind}}_{(f+v)}$, fixed and variable O&M costs of wind energy; $C^{\text{solar}}_{(f+v)}$, fixed and variable O&M costs of solar energy; C^{battery}_{f}, fixed O&M costs of battery systems; C^{battery}_{v}, variable O&M costs of battery systems; L, operating lifetime; S, project start time; C, construction time; $48E$, tax credit for renewable energy projects under the IRA; $45Y(T)$, tax credit for renewable energy production; M_{H_2}, hydrogen production flowrate; F_a, availability factor; L_{battery}, battery system lifetime; L_{wind}, wind farm lifetime; L_{solar}, solar panel lifetime; $CI_{\text{SMR}_{\text{EU}}}(T=0)$, carbon intensity of SMR in the EU at time $T=0$; $R_{\text{EU emissions}}$, emissions reduction rate in the EU; CO_2^{EUPrice}, EU carbon price; TPD, tonnes per day; $KgCO_2e$, kilograms of carbon dioxide equivalent; KgH_2, kilograms of hydrogen; MWh, megawatt-hour; kW, kilowatt.

The previous SI modules describe the model methodology for scenario A in its entirety. This section contains a set of case studies of this model. There are three dimensions to the case studies:

1. Business models: choice between scenarios A, B, and C. Scenario A is the baseline and is not discussed in this section.
2. CBAM: a choice on whether to enforce CBAM or not.
3. Electricity matching constraints: a choice to enforce yearly, monthly, or hourly constraints.

This section describes the differences between scenarios B and C from the baseline. We also describe the implementation of CBAM. Finally, we do not discuss matching constraints because the constraints are integrated into the optimization model (see electricity sources section).

Scenario-Specific Methods for Scenario B

To describe the changes we make to the baseline model (scenario A), we include a table with all the changes (supplementary table 35). We utilize some of the variables from the optimization problem and introduce some new variables in 33. We define the AP plant time step to be $T \in S+C+L$ months. Let $\Gamma = T \mod 12$ (so $0 \leq \Gamma \leq 11$). Γ is a useful transformation of T that allows us to extend the values of the optimization model from one year to 40 years of AP plant operation.

The decision between 48E and 45Y is found below (reference supplementary table 35):

Supplementary Table 35: Changes made to scenario A to make scenario C

**Change**	**Module Affected**	**Description**
Farm CAPEX	CAPEX	The CAPEX of each technology is increased by $COST_{\text{wind}} \times w + COST_{\text{battery}} \times b + COST_{\text{solar}} \times s$ except for AP SMR. The increased CAPEX is propagated to the rest of the CAPEX components.
Grid electricity costs	MDOPEX	Electricity costs from the grid are set to zero.
Farm OPEX	MIOPEX	The electricity costs are replaced by the variable and fixed O&M costs of the hybrid farm and battery system. $MIOPEX_{\text{el}}(\Gamma) = w \times \frac{C^{\text{wind}}_{(f+v)}}{12} + s \times \frac{C^{\text{solar}}_{(f+v)}}{12} + \left[\frac{b \times 1000}{12}\right] \times C^{\text{battery}}_{f} + d_{\text{monthly}}(\Gamma) \times C^{\text{battery}}_{v}$
$CI^{\text{Electricity}}_{(j \mid j \neq \text{SMR})}(T) = 0$	Carbon Intensity	We assume the carbon generated from the farm is zero.
48E or 45Y credits	Tax Credits	The NPV model has added tax credits coming from IRA programs that support the farm system. The decision between 48E and 45Y is based on the NPV of each cash-equivalent tax credit. The formulation of the decision is long, so we describe it in the scenario-specific methods
Selling surplus electricity	MDOPEX	Surplus electricity produced by the farm that is not stored can be sold to the grid at the higher price between the LCOE of the farm and the market price: $S_{\text{el}}(T) = [CF_{\text{wind}}(\Gamma) \times w + CF_{\text{solar}}(\Gamma) \times s - w_{\text{monthly}}(\Gamma) - s_{\text{monthly}}(\Gamma)] \times \max[LCOE_{\text{farm only}}, P_{\text{Electricity}}(T) \times 1000]$ So that $MDOPEX(T)$ is increased by $S_{\text{el}}(T)$.
Farm has replacement costs	MIOPEX	Every $L_{\text{solar}}, L_{\text{battery}}$, and L_{wind}, the uninstalled equipment cost of the assets is incurred. We make this assumption as the project lifetime of solar, wind farm, and battery storage is shorter than AP. The cost of the farm across time is the same cost as the start year. Hence, no technological improvements are taken into account. We adopt this assumption because the capacity factor data (and hence the optimization algorithm) is a function of the design parameters of the wind turbine. Relaxing this assumption brings about considerable computation barriers and also has little effect on the PPA price (scenario C) or NPV (scenario B) because the replacement cost is heavily discounted. Consider that 75% of the value of the NPV or PPA price is derived from the first 13 years of performance. At 20 years, the replacement cost plays a small part in the economic performance.

Abbreviations: CAPEX, capital expenditure; OPEX, operational expenditure; MDOPEX, market-dependent OPEX; MIOPEX, market-independent OPEX; $COST_{\text{wind}}$, wind farm cost per MW; $COST_{\text{battery}}$, battery system cost per MW; $COST_{\text{solar}}$, solar farm cost per MW; $MIOPEX_{\text{el}}$, additional OPEX due to electricity systems; $CI^{\text{Electricity}}_{(j \mid j \neq \text{SMR})}(T)$, carbon intensity of non-SMR technologies due to electricity; 48E, tax credit for renewable energy projects under the IRA; 45Y, tax credit for renewable energy production; $LCOE$, levelized cost of electricity; $P_{\text{Electricity}}(T)$, electricity market price at time T; $S_{\text{el}}(T)$, surplus electricity income; L_{solar}, solar farm lifetime; L_{battery}, battery system lifetime; L_{wind}, wind farm turbine lifetime; AP, ammonia production; SMR, steam methane reforming; NPV, net present value; PPA, power purchase agreement; IRA, Inflation Reduction Act.

$$\max\left[\mathbf{H}_{\text{CE}}\left(\frac{48E \times (\text{COST}_{\text{wind}} \times w + \text{COST}_{\text{battery}} \times b + \text{COST}_{\text{solar}} \times s)}{(1+d_r)^{S+C}}\right),\right.$$
$$\left.\sum_{T=S+C}^{L+S+C}\mathbf{H}_{\text{CE}}\left(\frac{(w(\Gamma)+s(\Gamma)) \times 45Y(T)}{(1+d_r)^{T-S}}\right)\right] \tag{62}$$

48E credits are redeemed at the start of operation and are discounted to the present value. We assume 48E credits also cover investments in the battery system.

45Y credits are spread out across time, hence the summation term. $45\text{Y}(T \mid S+C \leq T \leq S+C+E_{45\text{Y}})$ is non-zero and zero whenever $T \notin S+C \leq T \leq S+C+E_{45\text{Y}}$. The lifetime of 45Y credits, $E_{45\text{Y}}$, is determined to be when the grid emits 75% less than the 2022 grid. We first estimated this through $\frac{CI_{\text{AP\_AEC}}(T)}{CI_{\text{AP\_AEC}}(T=0)} = 0.25$ since $\frac{CI_{\text{grid}}(T)}{CI_{\text{grid}}(T=0)} \propto \frac{CI_{\text{AP\_AEC}}(T)}{CI_{\text{AP\_AEC}}(T=0)}$ because AP AEC's CI only varies with electricity. We find that grid emissions, according to the AEO2023, do not reach that level until after 2050[14]. For simplicity, we assume $2023 + \frac{E_{45\text{Y}}+S+C}{12} = 2050$ so that $E_{45\text{Y}}$ is a free variable that ensures $45\text{Y}(T)$ is zero after 2050.

Once a tax credit program is chosen, the tax credits are added to the total tax credits. In the case of 48E, 63 is used. 64 is used when 45Y is larger than 48E.

$$\text{Credits}_{\text{CE}_{\text{scenario B}}}(T) = \text{Credits}_{\text{CE}}(T) + \begin{cases} \mathbf{H}_{\text{CE}}\left[48E \times (\text{COST}_{\text{wind}} \times w \right. \\ \quad +\text{COST}_{\text{battery}} \times b \\ \quad \left. +\text{COST}_{\text{solar}} \times s)\right], & \text{if } T = S + C, \\ 0, & \text{otherwise.} \end{cases} \tag{63}$$

$$\text{Credits}_{\text{CE}_{\text{scenario C}}}(T) = \text{Credits}_{\text{CE}}(T) + \mathbf{H}_{\text{CE}}\left[(w(\Gamma)+s(\Gamma)) \times 45Y(T)\right] \tag{64}$$

Scenario-Specific Methods for Power-Purchase Agreement

The electricity price in scenario C becomes the LCOE of the hybrid farm, which we refer to as the PPA price. supplementary table 36 contains the details of how scenario C differs from scenario A.

Supplementary Table 36: Changes to the baseline model to enable scenario C. All changes apply to the low-carbon technologies. AP SMR stays the same as the baseline.

Change	Module Affected	Description
**Changes to the AP Model**		
Change in the electricity price	MDOPEX	The new electricity price corresponds to the LCOE of the hybrid farm.
**Changes to AP Model to derive the LCOE**		
Every probabilistic value becomes the average value	All modules	
Heuristic factors for the CAPEX are no longer multiplied by a factor of $\frac{1}{(2717/100)^{0.6}}$. (see section C)	CAPEX	This scaling factor for the heuristics is not valid for the hybrid facility because the facility does not produce ammonia.
All OPEX costs are substituted for heuristics	MIOPEX	The OPEX becomes the $MIOPEX(\Gamma)$. $MIOPEX_{\text{el}}(\Gamma) = w \times \frac{C^{\text{wind}}_{(f+v)}}{12} + s \times \frac{C^{\text{solar}}_{(f+v)}}{12} + \left[\frac{b \times 1000}{12}\right] \times C^{\text{battery}}_{f} + d_{\text{monthly}}(\Gamma) \times C^{\text{battery}}_{v}$
Sales change from ammonia to electricity	Sales	$Sales(\Gamma) = [D_j + (CF_{\text{wind}}(\Gamma) \times w + CF_{\text{solar}}(\Gamma) \times s - w_{\text{monthly}}(\Gamma) - s_{\text{monthly}}(\Gamma))] \times LCOE$
Only 48E or 45Y credits are considered	Tax Credits	48E or 45Y credits are applied using the same formulation as scenario C (see section F.1.1).
Instead of an NPV analysis, we use a levelized cost analysis	DCF Model	We iterate the LCOE until the absolute value of the NPV is minimized. $\arg\min_{\text{LCOE}} \sum_{T=0}^{L+S+C} \left\lvert \frac{CF_{\text{LCOE}}(T)}{(1+d_r)^{T-S}} \right\rvert$

Abbreviations: AP, ammonia production; SMR, steam methane reforming; LCOE, levelized cost of electricity; CAPEX, capital expenditure; OPEX, operational expenditure; MDOPEX, market-dependent OPEX; MIOPEX, market-independent OPEX; $MIOPEX_{\text{el}}$, electricity-related OPEX; $CF_{\text{wind}}(\Gamma)$, wind capacity factor; $CF_{\text{solar}}(\Gamma)$, solar capacity factor; w, installed wind capacity; s, installed solar capacity; b, installed battery capacity; $C^{\text{wind}}_{(f+v)}$, fixed and variable O&M costs of wind farms; $C^{\text{solar}}_{(f+v)}$, fixed and variable O&M costs of solar farms; C^{battery}_{f}, fixed O&M costs of battery systems; C^{battery}_{v}, variable O&M costs of battery systems; $d_{\text{monthly}}(\Gamma)$, battery discharge; $w_{\text{monthly}}(\Gamma)$, monthly wind generation; $s_{\text{monthly}}(\Gamma)$, monthly solar generation; 48E, tax credit for renewable energy CAPEX under IRA; 45Y, tax credit for renewable energy production; NPV, net present value; DCF, discounted cash flow; d_r, discount rate.

The levelized cost method applied to the hybrid farm system initially follows the same method as the baseline AP NPV model, with an additional step to minimize the absolute value of the NPV by iterating through the levelized cost (LCOE or PPA price).

Scenario-Specific Methods for Carbon Border Adjustment Mechanism

The implementation of CBAM is a simple formula that measures the difference between the European AP SMR CI and the technology j. The magnitude of the difference is translated into the carbon tax below:

$$\text{CO}_2\text{tax} = (CI_{\text{SMR\_EU}}(T) - CI_j(T)) \times M_{H_2} \times \frac{365}{12} \times F_a \times \text{CO}_2\text{EUPrice} \quad (65)$$

$$CI_{\text{SMR\_EU}}(T) = CI_{\text{SMR\_EU}}(T=0) \times (1 - R_{\text{EU\_emissions}})^{T/12} \quad (66)$$

We assume CBAM does not expire. The values for each parameter are found in **??**. We calibrated $R_{\text{EU\_emissions}}$ so that the EU AP SMR emissions in 2050 will match the predictions by the European Commission and Kakoulaki et al.[30,31]. We chose an exponential decay type of relationship to show more pronounced emissions reductions early in the century and slower reductions towards the middle of the century.

Supplementary Discussion

This section briefly reviews the four AP technologies this report considers. supplementary figure 4 shows a visual schematic of the technologies analyzed in our study. We start with the carbon-intensive, conventional AP SMR. Then, we describe AP SMR with Carbon Capture and Storage (AP CCS), followed by AP SMR with carbon-neutral biomass, as feedstock (AP BH2S). The last low-carbon technology pathway we considered for the AP is via Alkaline Electrolysis (AP AEC). Additionally, we include a comparison of each AP technology's technical advantages and disadvantages.

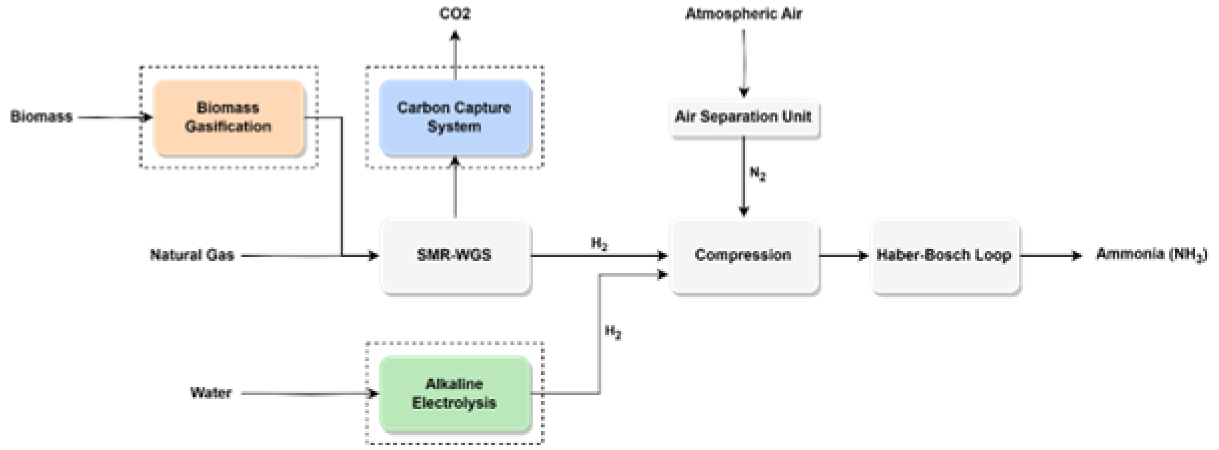

Supplementary Figure 4: Technology pathways analyzed in this article. This figure illustrates the technology pathways for ammonia production, including steam-methane reforming (SMR) and water-gas shift (WGS) processes. Abbreviations used: SMR stands for steam-methane reforming, and WGS stands for water-gas shift.

AP through Steam Methane Reforming (baseline scenario)

The common denominator across all technologies is the notable Haber-Bosch (HB) process – a chemical process by which pure sources of hydrogen (H_2) and nitrogen (N_2) gases combine to form ammonia (NH_3). The source of N_2 is separated from the air with an air separation unit (ASU), as air is 78% N_2 by volume. For H_2, natural gas (NG) is the most prominent source (78% of global AP) – although coal is also common (22%)[32]. NG is chemically treated to obtain pure H_2 for the HB process selectively. Specifically, the chemical treatment for hydrogen production has three major steps[1,33–35]:

1. NG is cleaned of impurities such as sulfur (Kim et al., 2021).
2. The NG is mixed with steam and reacted in several heated vessels – the first reaction is called steam-methane reforming (SMR), and the second is the water-gas shift (WGS) reaction. The resulting chemicals from these reactions are methane, CO_2, and H_2.
3. H_2 is separated from CO_2 and methane in a pressure-swing absorption unit (PSA). The pure H_2 product is sent to the HB process. The remaining methane and CO_2 heat the SMR vessel through combustion. Finally, the combusted gas is emitted into the environment through the plant's stack.

The H_2 from the HP process and the N_2 from the ASU must be compressed because the HB process requires extreme pressures and temperatures. The compression of H_2 and N_2 is the most energy-intensive step in AP. The source of energy for compression varies across technologies (see the technical supplementary methods section).

The typical AP SMR plant produces 500 to 3000 metric tons per day (TPD) of NH_3 [1,36]. The IEA reports that the break-even AP SMR ammonia price ranges from approximately $300 to $600 per ton of NH_3, whereas the market price is between $200 and $750 in 2021 [36]. Natural gas price constitutes 30% of the levelized cost of ammonia (LCOA) as it is needed for hydrogen production and heating [33,37,38]. Therefore, a strong correlation between natural gas and ammonia markets exists. The remaining cost is attributed primarily to capital expenditure (CAPEX) and the rest to operational expenditure (OPEX) [33,37].

AP SMR with a Carbon Capture System (AP CCS)

The state-of-the-art CCS technologies are amine-based carbon sequestration units operated commercially for direct air or point-source capture. This CCS technology operates by mixing CO_2-rich gases with water and amine solution to dissolve the CO_2 in the solvent. The CO_2-rich solvent can be stripped of the CO_2 by heating – effectively regenerating the solvent and obtaining pure CO_2 gas for transportation and storage. CCS systems can capture up to 95% of the CO_2 within the AP plant at an additional electric energy penalty cost relative to AP SMR. A report by the DOE comparing hydrogen production via SMR with and without CCS found that H_2 SMR required 0.65 kWh per Kg H_2 of electricity, while H_2 SMR with CCS required 2.04 kWh per Kg H_2 [2].

AP SMR with a Biomass-derived feedstock (AP BH2S)

The natural gas feedstock of AP SMR can be substituted with biomass. Hydrogen production with a biomass feedstock utilizes the organic compounds in the biomass to generate small gaseous molecules (i.e., CH_4, C_2H_4, CO, CO_2, H_2, N_2, etc.) through a process known as gasification. These molecules are further processed into H_2 and CO_2 through the conventional SMR-WGS steps [39–41]. According to Spath et al., the organic molecules are converted to small molecules in a separate tar reformer before the SMR to pre-treat the syngas for sulfur contaminants and avoid char formation – although the goal design would be to perform tar reforming and SMR in the same vessel [6].

Biomass feedstocks are effectively net-zero, as the carbon emitted by biomass comes from the atmospheric CO_2 fixated into plants through photosynthesis. However, the electric energy requirements to process the biomass into usable NG-like synthesis gas are in the ballpark of AP CCS [2,6]. Hence, significant and possibly IRA-disqualifying indirect emissions of AP BH2S may be a fault of a carbon-intensive electric source (i.e., the electric grid) [14].

AP via Alkaline Electrolysis (AP AEC)

The most abundant source of hydrogen is water. Water contains zero carbon and can be electrolyzed to produce pure hydrogen and oxygen gas. The advantage of electrolysis is that it is modular and suitable for decentralized systems [7,9]. The current competing technologies are alkaline water electrolysis (AEC), proton exchange membrane electrolysis (PEM), and solid oxide electrolysis (SOE) [7,8,42,43].

AEC is the most competitive technology as it has a high lifetime (60,000-90,000 hours (h)) and a low cost (1300-500 $/kW) when compared to PEM (20,000-90,000h and 2000-800 $/kW) [8]. SOE stacks have the highest cost among all technologies (5000-1500 $/kW) and the shortest lifetime (¡20,000h) as they are still at the laboratory stage [8]. Nevertheless, SOE stacks are expected to experience the most significant cost reductions from deployment and R&D [8,44]. AP AEC forms a small part of AP in general (¡0.02 Mt in 2021) [7,34]. AEC pathways and other electrolysis technologies expect cost reductions of 0-24% from R&D and 17-30% from production scale-up [8].

While electrolytic pathways mitigate emissions by ensuring a zero-carbon feedstock, the additional electric energy demand can be higher [45]. Gomez and colleagues identified that H_2 electrolysis required 50-60 kWh per kg H_2 versus 0.65 kWh per kg H_2 for SMR [2,46]. The additional electricity usage renders electrolytic hydrogen production to be non-zero because of the indirect emissions of the grid and construction materials.

Simons and Bauer estimate solar-powered electrolysis at 3 kg CO_2 eq per kg H_2 and wind at 2 kg CO_2 eq per kg H_2 due to the indirect emissions of the construction materials for wind and solar alone[47].

Economic and environmental comparison of AP across the literature

The most often used and preferred method in the literature to compare economic performance of low-carbon hydrogen and ammonia is the levelized cost approach (LCOH and LCOA)[2,9,38,40,41,44,46,48–55]. The LCOA, while pertinent in numerous instances, is primarily focused on the production cost side of the equation, potentially overlooking the IRA's significant dynamics, which influence the revenue side.

This revenue-side influence, particularly concerning income taxes, is outside the scope of the LCOA, necessitating assumptions that could potentially overstate outcomes. For instance, Jenkins et al. implicitly assumed all tax credits equal \$1 US dollar by awarding full credit value to their levelized cost analysis[56]. This approach may lead to overestimations, particularly regarding policy support for low-profit, riskier, low-carbon technologies that heavily rely on a tax credit market. Consequently, this study adopts the NPV approach, offering a more comprehensive and nuanced perspective better suited to capturing the real-world effects of income tax credit-based policies like the IRA.

Regarding the electric energy intensity of AP, compressing the gas out of the HP and ASU systems before the HB loop is known to be a highly energy-intensive step. Hence, using surplus energy from other parts of the process is a critical step that may sometimes drive AP SMR to generate electricity[5]. This energy integration step is essential in determining the relative OPEX between AP SMR and low-carbon technologies. Some technologies lack surplus energy to power the compression and need to purchase energy from the grid.

In general, AP SMR may have surplus energy to power the entire compression load, and hence, it uses the least amount of grid electricity[2,5]. AP CCS does not have surplus energy and uses grid electricity[2,5]. AP BH2S does have surplus energy for one compression process but has two in total—hence using around the same amount of grid electricity as AP CCS[6]. Finally, AP AEC needs an order of magnitude larger amount of electricity for hydrogen production. It does not have surplus energy for compression—making it the most electrically energy-intensive process in the portfolio[46]. Regarding total energy efficiency, AP AEC is the least efficient, followed by BH2S and CCS. AP SMR is the most energy-efficient pathway[6,40,50,57].

In terms of economic cost assessment, large-scale AP SMR plants, with capacities above 2000 TPD of ammonia, can have capital expenditure (CAPEX) ranging from \$500M (\$250k per TPD NH_3) to \$1800M (\$900k per TPD NH_3) and operating expenditure (OPEX) ranging from \$180 to \$500 per ton of NH_3[36,37,58]. For AP CCS at an 88.2% capture rate, the CAPEX is estimated to be between \$298k per TPD NH_3 and \$275k per TPD NH_3, with an OPEX of €280 per Ton NH_3[50]. On the other hand, AP BH2S costs have been studied at scales of 73 to 1187 TPD NH_3. Arora et al. provided a detailed process model for biomass gasification at 73.5 TPD NH_3[39]. They noted that the CAPEX and OPEX of biomass gasification are between \$170k to \$175k per TPD NH_3 and \$705 to \$722 per ton NH_3, respectively.

Although specific cost data for AP AEC was not easily found in the literature, it is likely to have similar or higher costs than AP CCS and AP BH2S, depending on variable factors such as electricity costs (OPEX-related) and electrode stack costs (CAPEX-related), which are highly uncertain variables[9]. AP AEC is the pathway expected to reduce cost due to modularity[8]. AP CCS is expected to remain at a similar cost level—hence only seen as a transitory technology for decarbonization[35]. AP BH2S, on the other hand, presents significant uncertainties regarding its cost and feedstock availability[40,41,54].

On the environmental front, life cycle assessments (LCA) of AP SMR produce variable results involving the emissions intensity of ammonia production. The cradle-to-gate equivalent CO_2 emissions of AP SMR varied by 10 to 15% from the average across studies[59–61]. For example, Bicer and colleagues measured the emissions intensity for AP SMR to be 1.6 kg of equivalent CO_2 emissions (kgCO_2e) per kg of NH_3 with a plant-wide scope, while ARPA-E reported a value of 2.55 kgCO_2e per kgNH_3 for a cradle-to-gate analysis using the GREET model[59,60]. Liu and colleagues reported emissions intensities of around 1.8 kgCO_2e per kgNH_3 for a cradle-to-gate scope[61]. Young and colleagues found that CCS reduced cradle-to-gate CO_2e emissions by 69%[62].

The DOE report on hydrogen production via SMR with CCS found similar results at a 61% reduction in cradle-to-gate CO_2e intensity[2]. The average CO_2e intensity of AP SMR across four studies is approximately 11.7 kgCO_2e per kgH_2 (1.99 kgCO_2e per kgNH_3)[59–61] – Please note that reductions by 61-69% qualify AP SMR with CCS for significant 45Q carbon sequestration credits under IRA[2,23,63].

AP through BH2S has been considered a viable alternative to ammonia production as it is a zero-carbon fuel[39,48,64]. Gilbert and colleagues show that biomass reduces cradle-to-gate emissions to 0.7 kg CO_2e per kg NH_3 (3.95 kg CO_2e per kg H_2) from a 1.9 kg CO_2e per kg NH_3 (10.7 kg CO_2e per kg H_2) natural gas AP SMR baseline[64]. The environmental performance of AP w/ BH2S has also been shown to decrease with increasing scale at varying proportions depending on the type of biomass. Arora et al. display results indicating the inverse relationship between decreasing life-cycle emissions and scale-up cost reductions[39].

While AP AEC pathways mitigate emissions by ensuring a zero-carbon feedstock, the additional indirect electric energy emissions can be higher. The estimated potential emissions intensity of AP AEC can range between 4.4-2.2 kg CO_2e per kgH_2. Simons and Bauer estimate solar-powered electrolysis at 3 kg CO_2e per kgH_2 and wind at 2 kgCO_2e per kgH_2 due to the indirect emissions of the construction materials for wind and solar[47]. Borole and Greig estimated wind-powered electrolysis at 0.97 kgCO_2e per kgH_2, and Valente et al. estimated 0.3 kgCO_2e per kgH_2[65,66]. Liu and colleagues estimated the emissions intensity of N_2 production and the Haber-Bosch process to be 0.3 kgCO_2e per kgH_2 and 0.9 kg CO_2e per kgH_2, respectively[61]. By adding Liu et al.'s estimates to the results, the estimated potential emissions intensity of AP AEC can range between 4.4-2.2 kg CO_2e per kg H_2.

In essence, AP CCS and AP BH2S are effective strategies for significantly reducing the carbon emissions associated with AP SMR. Despite this, AP CCS is inherently limited by a capture rate that falls short of 100%, leading to unavoidable residual emissions. In contrast, AP BH2S holds the potential for near-net-zero emissions at the risk of limited feedstock supplies and quality. Alternatively, AP AEC can attain net-zero emissions, provided that the emissions linked to the production of materials are disregarded, especially in scenarios where the power generation is green.

Haber-Bosch Flexibility

The maximum flexibility of the HB loop varies widely between sources, ranging from 10% of nominal capacity[67] to nearly 80%[68], with most estimates being concentrated around the 40% level. This wide variance can be attributed to a range of underlying assumptions made by various authors; Verleysen et al. conservatively estimate a flexibility of 78.7% of maximum capacity while optimizing system performance under realistic operational constraints[68]. They also observe a significant 76.2% decrease in mean AP for a 9.33% increase in flexibility.

Another study by Cheema and Krewer found through physicochemical modeling that by reducing the H_2-N_2 ratio in the feed, H_2 consumption can be reduced by 67% with a consequential 17% increase in recycle load[69]. Cocon conducted a technical design optimization of an HB reactor while fluctuating the feed stream composition (H_2:N_2) from 3:1 to 1.31:2.69 over a 24-minute simulation. No substantive comments were made on the economic implications of implementing such a reactor[70].

Armijo and Philibert outline a standard flexibility case through interviews with manufacturers where HB flexibility is 40% of nominal capacity with a ramp rate of ±20% of nominal capacity per hour[71]. Further, they conclude that this ramp rate is more than sufficient to not be a limiting factor, which is corroborated by Wang et al.[72]. Another first-principle-based analysis by Lazouski et al. finds that the energy efficiency of the ammonia production reaction must be greater than 32%, and energy efficiencies below 30% dramatically increase energy costs[73].

These studies primarily examine the technical capabilities of HB while forgoing economic feasibility evaluation. Analyses based on cost-effectiveness yield a smaller operable capacity range; a study by Onodera et al. found a cost-optimized capacity factor for HB of 73% as part of a larger flexible AP system. Further, they found that a flexible production capacity was economically preferable to a battery buffer storage system[74].

Allowing for flexible HB operation involves weighing the costs and benefits of reduced electricity matching requirements against the obvious reduced production, but also the increased risk of damaging process equipment under non-optimal operating conditions[67]. Flexibility in HB operation is a prominent cost driver as the CAPEX associated with supplementary renewable energy infrastructure is significantly larger than that of ammonia production or the generation of hydrogen. Oftentimes, the minimum load of a flexible HB process is more impactful on overall process system costs than HB CAPEX itself given the impact of minimum load on the sizing of supplementary power equipment[72]. It was determined that a minimum HB load decrease from 60% of nominal to 10% supported by a hybrid renewable energy system (wind and PV) resulted in a 7.1% and 3.9% decrease in LCOA in two Australian locations for which simulations were

conducted[72]. Bose et al. study costs of a dynamic green ammonia production plant with green energy[75]. They take into account the spatio-temporal variation of VRE resources. Bose et al. find that 50% capacity flexibility in HB results in a 15% decrease in LCOH[75]. However, this LCOH is not competitive with the incumbent grid-connected technology ($400 per t vs. $800 per t). Bouaboula et al. performed a techno-economic analysis of a PEM AP system with storage to address steady-state HB constraints. The resulting LCOA in 2020 is $780 per t, 2030 is $400 per t, and 2050 is $250 per t. LCOA reductions originate from solar, wind, and electrolysis equipment becoming more competitive. Like our study, Bouaboula et al. find the cost reductions are non-linear with time[76].

While flexibility in an HB loop is possible, research into its practical implementation and cost-effectiveness is in its early stages. The breadth of literature suggests the extent to which the HB process can be operated flexibly depends on the lens through which analysis is conducted. Multiple technical analyses and patents purport HB operating ranges down to 10% of nominal capacity with ramp rates of ±20% per hr[67]. That said, economic analyses find optimal performance within significantly more stringent constraints (between 70% and 80% capacity)[68,74].

There are groups working to better understand the state of flexible HB technology through more pragmatic implementation. A report for the British government introduces the Ammonia Synthesis Plant from Intermittent Renewable Energy (ASPIRE) project. This project proposes the use of seven synthesis reactors in parallel which can individually be powered on and off to achieve flexibility of down to 5% of maximum capacity (0.5MW to 10MW), storing excess thermal energy to keep idle reactors up to operating temperature[77]. They also find that flexible HB design is preferable to either energy or hydrogen storage units due to significantly reduced CAPEX. They claim that the technology for this flexible design is commercially available, but even their model plant is still in the planning stages.

The Danish green energy company Topsoe plans to pilot a 25 MT per day ammonia plant with 10% to 100% flexibility in early 2024, with further plans to upscale to 1800 MT per day by 2025[78]. Even when process economics are considered, there is little research into how incorporation of flexibility into an HB process will affect CAPEX, making the overall value of such a development difficult to estimate. Even Topsoe acknowledges cost optimization has not been performed on the pilot plant due to small-scale process economics not being reflective of full scale. With the IRA expiring in 2035, there are economic incentives to utilize mature, available technologies to maximize the benefits of these policies[78].

Convergence of Monte Carlo results

This study analyzed the variability of NPV values across total Monte Carlo simulations for SMR, CCS, BH2S, and AEC technologies. The primary objective was to assess the robustness of NPV values as they evolved with an increasing number of simulations, offering insights into their stability in response to varying levels of simulation intensity.

For each technology, scenario, and year, we conducted simulations with 500, 1,000, 2,000, 4,000, and 8,000 iterations (see the annexed Excel file named *AP_NE_Convergence_On_NPV.xlsx*). During these simulations, we collected statistical data, including maximum, minimum, P95, P5, mean, and median values. Based on these results, we selected 4,000 simulations as the optimal number for data collection, considering the balance between convergence and computational efficiency.

At the beginning of our analysis, significant fluctuations in NPV values were observed during the initial 300 iterations, indicating a potential uncertainty in early simulations. However, beyond approximately 2,000 runs, NPV values began to stabilize, with fluctuations diminishing substantially. Across most scenarios, a clear convergence trend emerged after the 2,000-run mark, suggesting that further iterations had a minimal impact on NPV values. We determined that 4,000 simulations provided the right balance between ensuring convergence and maintaining computational tractability.

Comparison of SMR baseline to IEA

The IEA provides a detailed chart of a levelized cost analysis performed on various technologies. To validate our techno-economic methodology, we compare our AP SMR levelized cost to the AP SMR LCOA reported by the IEA (Figure 1.6, page 40) with the deterministic levelized cost from this study[36].

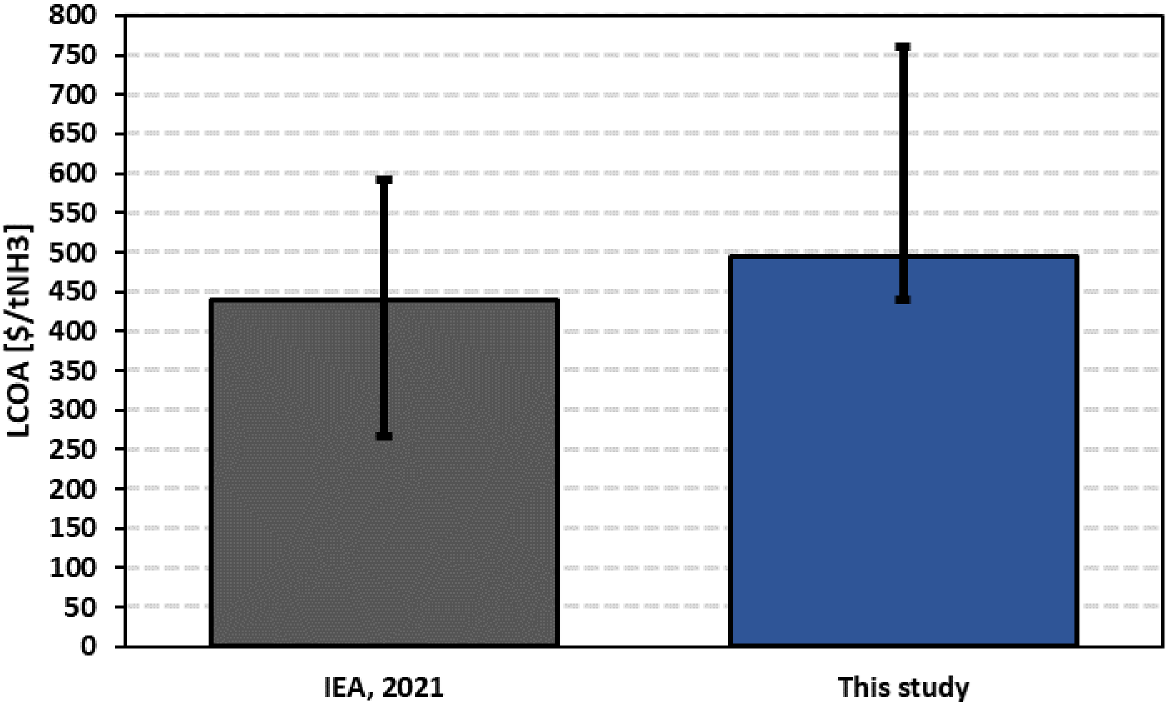

Supplementary Figure 5: Deterministic levelized cost of ammonia technologies. This figure compares the deterministic levelized cost of ammonia from this study to the International Energy Agency's (IEA) Ammonia Technology Roadmap report[36].

We used the sensitivity inputs for natural gas and electricity from the IEA to obtain the range of LCOAs, as shown in supplementary table 37. The IEA assumes a higher capacity factor of 95% and smaller CAPEX heuristics, resulting in a reduced CAPEX compared to our study. supplementary figure 5 below illustrates this comparison. Additionally, the IEA assumes a smaller discount rate of 8% (compared to our 9.3%) and a shorter operating lifetime of 25 years versus 40 years. Consequently, their TEA is less sensitive to changes in OPEX due to the shorter operating lifetime, though the difference in discount rates may offset this effect.

The deterministic LCOA is computed by setting the NPV to zero (similar to the LCOE, see SI D). The deterministic NPV is calculated by taking the average of all probabilistic inputs. The high and low error bars represent instances when the model takes the maximum and minimum probabilistic values, respectively. Furthermore, in the minimum and maximum scenarios, we take on the electricity and natural gas values that the IEA uses (see supplementary table 37).

In conclusion, the values we obtain are comparable to those reported by the IEA, despite the significant differences in input values. Our plant model shows greater sensitivity to fluctuations in natural gas, ammonia, and electricity markets, denoted as $P_i(T)$ in the supplementary methods section, than the IEA's AP SMR model. Additionally, our conservative estimation of CAPEX, incorporating additional cost factors, leads to an upward shift in the LCOA compared to the IEA's LCOA.

Sensitivity analysis of inputs

Ensuring the quality of our TEA through a sensitivity analysis will help us determine if directional changes in the inputs result in economically sensible shifts in the NPV of the technologies. We include an Excel file named *AP_NE_Sensitivities.xlsx*, which shows the difference in NPV [$/tNH$_3$] between the deterministic baseline and the sensitivity analysis. The "input" column depicts the change in the input variables in the high and low scenarios. The sensitivities are ranked in descending order by the sum of the NPV shifts across

technologies.

We set the bounds of the sensitivity analysis to be the distribution limits for probabilistic values and ±20% for deterministic and composite values. Composite values are values that sit in between the outputs and inputs. For example, the OPEX is a composite value because it is not an input or an output.

There are values in the file that do not contain the input bounds for the sensitivity analysis – namely, "AEC Stack Cost" and "Wind Turbine CAPEX." This is because the bounds of these values change over time. To find the bounds, refer to the 2023 and 2030 costs we set for these costs in SI B and D.

In the deterministic version of the AP model, we handled commodity prices differently from the probabilistic counterpart. We set the standard deviation of the GBM module to 0 to express linear changes in commodity prices across time. This slightly affects the sensitivity analysis results.

CAPEX-related sensitivities

We consider the AEC CAPEX, the total CAPEX, and the wind CAPEX for the sensitivity analysis. We do not consider the battery CAPEX since monthly matching has no allocated battery capacity.

For validation, the wind and battery CAPEX have the same sign (negative) and are part of the same supplementary equation in the Python model. Hence, they behave similarly in the hourly matching sensitivity (where battery capacity is allocated).

Varying the overall CAPEX (composite value) by ±20% has little effect on the NPV in scenario A. Less than \$1 per tNH_3 was recorded, so it was rounded to 0, as such a difference is statistically insignificant. In scenario B, the CAPEX disproportionately affects the AP CCS, AP BH2S, and AP AEC NPVs relative to AP SMR. This is caused by the additional cost of the hybrid wind farm, which increases the CAPEX for the low-carbon technologies. Consequently, changes by ±20% in the low-carbon CAPEX will also vary the CAPEX of the hybrid wind farm – thereby becoming a more sensitive parameter. AP AEC is the most sensitive because its CAPEX is the biggest (see *AP_NE_CAPEX.xlsx*). In scenario C, the CAPEX sensitivity regresses to scenario A sensitivity because the only difference between scenario A and scenario C is the OPEX.

This parameter is more sensitive than the CAPEX for the AEC stack CAPEX. This is because the range of variation, in terms of percentage points, is ±33%. Remember that the AEC stack CAPEX is a probabilistic parameter that varies according to its bounds.

OPEX-related sensitivities

We consider the process-related OPEX (MI OPEX) and market-dependent commodity prices (electricity and natural gas) for monthly matching.

The process-related OPEX is the largest in scenario B because of the O&M costs of the hybrid wind farm for low-carbon technologies and appears correlated with the electricity price. AP SMR only experiences a shift of \$6 per tNH_3 when decreasing the process-related OPEX by 20%. Meanwhile, AP AEC experiences a change of \$65 per tNH_3 with the same change. This is due to the scale of the hybrid wind farm, which incurs severe O&M costs. Note that the hybrid wind farm scale is directly proportional to the electricity demand of the AP technology. AP CCS and AP BH2S sit between AP SMR and AP AEC, with changes of \$19 per tNH_3 and \$25 per tNH_3 in scenario B, respectively.

In scenarios A and C, the process OPEX is similar for all technologies. This is because the electricity price is not part of the process OPEX—the electricity price being the only difference between scenarios A and C in terms of OPEX. Hence, the cost drivers cause the same directional change. This change is less than in scenario B. AP SMR, AP CCS, AP BH2S, and AP AEC only incur shifts of \$6 per tNH_3, \$8 per tNH_3, \$15 per tNH_3, and \$9 per tNH_3 with a 20% decrease in the process OPEX, respectively.

Natural gas and electricity prices were found to be significant cost drivers of the AP process. In the probabilistic model, we enforce a correlation between ammonia and natural gas prices through a bivariate distribution. In the deterministic model, we take the average markup from natural gas to ammonia prices and make the ammonia price dependent on the product of the natural gas price and the markup. We do this to capture the hedging effect observed in the probabilistic model.

The natural gas price alone drives a large part of the NPV of AP SMR and AP CCS across all scenarios. We set a range of prices by taking the minimum and maximum natural gas prices from December 2014 until

January 2023 (EIA, 2023b). Changing the natural gas price results in hedging, as seen when the price is set at $2.58 per MMBtu. The NPV of AP SMR and AP CCS decreases by -$42 per tNH_3 and -$48 per tNH_3, while AP BH2S and AP AEC decrease by -$75 per tNH_3 and -$84 per tNH_3. At the high sensitivity ($9.95 per MMBtu), the NPV of AP SMR and AP CCS decreases by $90 per tNH_3 and $91 per tNH_3, while AP BH2S and AP AEC decrease by $100 per tNH_3 and $109 per tNH_3. The hedging effects result in approximately twice the loss for non-hedged technologies (AP BH2S and AP AEC) when natural gas prices reduce (and hence ammonia prices). On the other hand, the potential gain from not hedging results in a 21% higher increase in NPV for BH2S and 11% for AP AEC.

The markup between NH_3 and natural gas was also varied. The low is 58.96 $$NH_3$ per $NG and the high is 204 $$NH_3$ per $NG. Changing the markup directly changes the ammonia price without affecting the natural gas price. Consequently, both AP SMR and AP CCS increase by $51 per tNH_3 when the markup is high. Similarly, AP BH2S and AP AEC increase by $52 per tNH_3 and $62 per tNH_3. On the other hand, low markup results in a loss of -$76 per tNH_3, -$86 per tNH_3, -$85 per tNH_3, and -$94 per tNH_3 for AP SMR, AP CCS, AP BH2S, and AP AEC, respectively. These results vary slightly across scenarios due to tax-credit and profitability effects (see section I). Nevertheless, the general trend holds.

The electricity price is one of the foundations of the NPV difference between AP SMR and the low-carbon technologies (especially AP AEC). In scenario A, the electricity price affected AP SMR, AP CCS, and AP BH2S less than their feedstock cost—specifically by +/- $1 per tNH_3, $3 per tNH_3, and $3 per tNH_3, respectively. The effects of biomass feedstock on BH2S were +$14 per tNH_3 and -$15 per tNH_3 for a decrease to $50.68 per dry tonne and an increase to $118.24 per dry tonne, respectively. Meanwhile, AP AEC uses electricity to drive the energy input into the hydrogen product (given the delta in thermodynamic energy states of water and hydrogen gas), which results in a dramatic sensitivity of +/- $29 per tNH_3 for a +/- 20% change in the electricity price in scenario A.

In scenario B, the electricity cost sensitivity goes to zero for the low-carbon technologies and remains at the same level for AP SMR. This is because the electricity costs are shifted to the hybrid wind farm CAPEX and O&M costs.

In scenario C, the electricity cost sensitivities are the same as in scenario A. Although AP AEC shows slightly less sensitivity at +/- $28 per tNH_3, this difference can be considered negligible.

Policy sensitivities

We consider sensitivity of ±20% for all programs except 48E, which was varied between 30% and 40% ITC. In scenario A, 45V credits are the only sensitive parameter. AP CCS can claim 45Q – however, this choice is suboptimal. In scenario A, AP CCS and AP BH2S are the only technologies receiving tax credits. Changing 45V credits by +20% results in a change of $4 per tNH_3 and $3 per tNH_3 for AP CCS and AP BH2S, respectively.

In scenario B, 45V and 48E credits are the only sensitive parameters. 48E credits are always preferred over 45Y credits given that the return on energy per $ CAPEX invested in the wind farm is not high enough to make 45Y credits desirable. We find that 45V credits have a symmetric and similar effect for all three low-carbon AP technologies. The variation is ± $12 per tNH_3 for AP CCS, AP BH2S, and AP AEC. 48E credits are symmetrical around the high and low sensitivities but not across technologies. The high electricity demand of AP AEC increases the scale of the hybrid wind farm – thereby increasing the amount of 48E credits awarded to AP AEC. We see AP AEC net an increase in NPV of ± $13 per tNH_3. AP CCS and AP BH2S only receive ±$2 per tNH_3. Note that for the deterministic model, we took the inputs as the average of the probabilistic ranges, hence the baseline value for 48E credits is 35% – which is technically not possible given the step-wise nature of the 48E program (either 30% or 40%). This discontinuity is expressed in the probabilistic model. If we were to set the 48E credits to 30%, then the sensitivity would be $0 per tNH_3 in the low scenario and twice the change in the high scenario.

In scenario C, 45V credits have the same effect on the NPV as in scenario B. In scenario C, all other credits are not sensitive because (i) the hybrid wind farm is not part of the system and (ii) 48Q credits are not preferable to 45V credits in the case of AP AEC – these two conditions leave only 45V credits.

Conclusion on the sensitivity analysis

In conclusion, we find sensical changes in the NPV when varying the inputs. First, cost-side variables drive the prices down, and revenue-side variables drive prices up. Second, in areas related to asymmetric resource demand across technologies (i.e., electricity demand), we see unequal changes in the NPV per unit input. On the revenue side, we find the expected hedging that AP SMR and AP CCS have against ammonia and natural gas price movements. In cases where the carbon intensities are high, we see a decreased sensitivity in policy support – which is also expected. With these results, we find confidence in the formulation of the model.

717 # Supplementary Figures

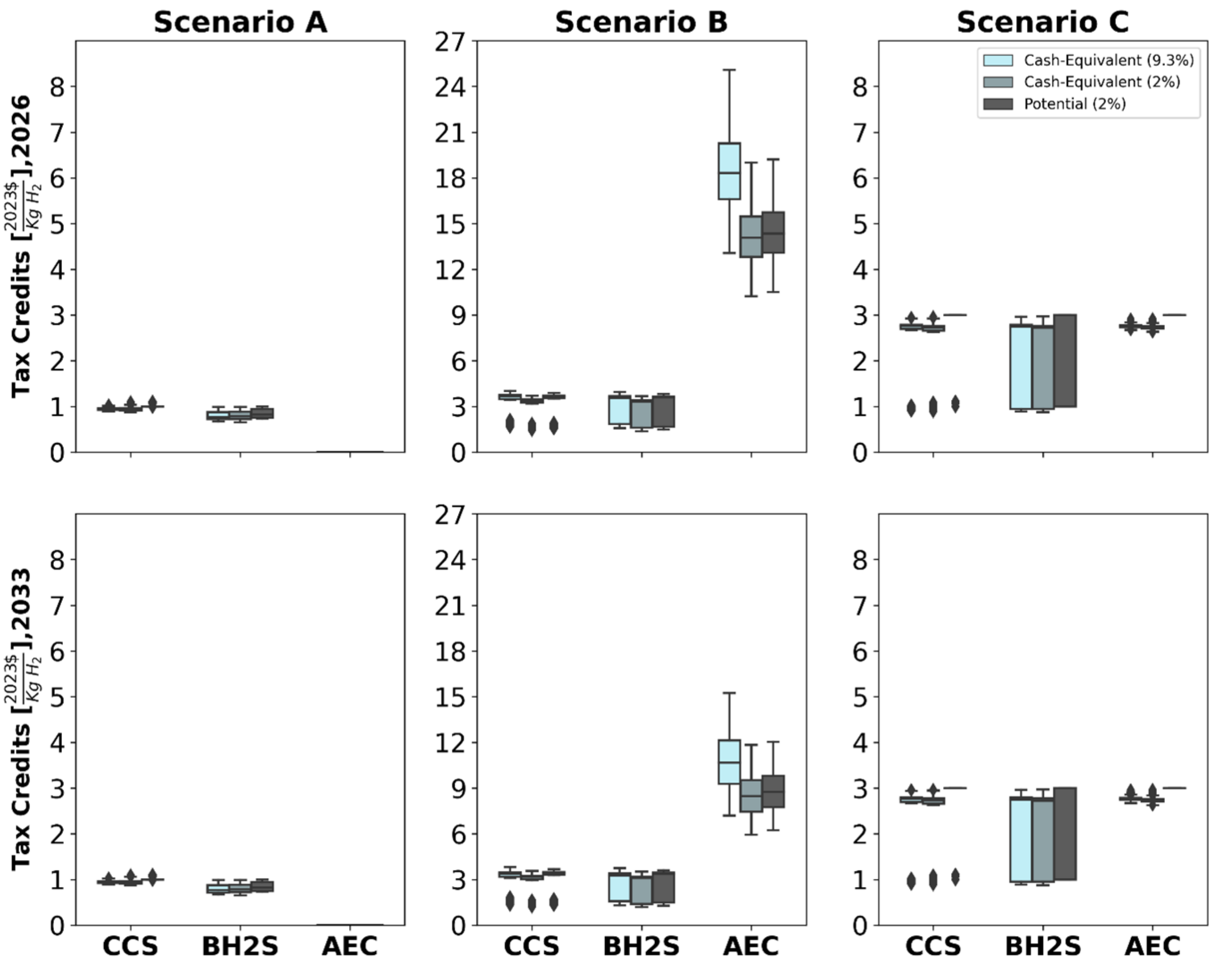

Supplementary Figure 6: Hourly matched total policy support for ammonia technologies. This figure compares the total policy support (in \$ per kg H_2) provided for various low-carbon ammonia production technologies across two main scenarios—Scenario A and Scenarios B/C—and two different electricity configurations: grid-based electricity and hybrid renewable electricity farms. Scenario A accounts for policy support related to emissions from the stack, feedstock, and electricity sources, while Scenarios B and C assume policy support for stack and feedstock emissions only, under the assumption of zero emissions from renewable energy sources. CCS refers to ammonia production with carbon capture, BH2S refers to ammonia production with biomass gasification, and AEC refers to ammonia production through alkaline electrolysis. The light blue boxes signify cash-equivalent tax credits discounted at 9.8%, which are credits adjusted for income tax. The darker blue boxes represent the same tax credits discounted at the risk-free rate, while the gray boxes denote the same tax credits without accounting for income tax. Note that the Scenario B charts have a y-axis three times larger than those of Scenarios A and C to accommodate the range of policy support values.

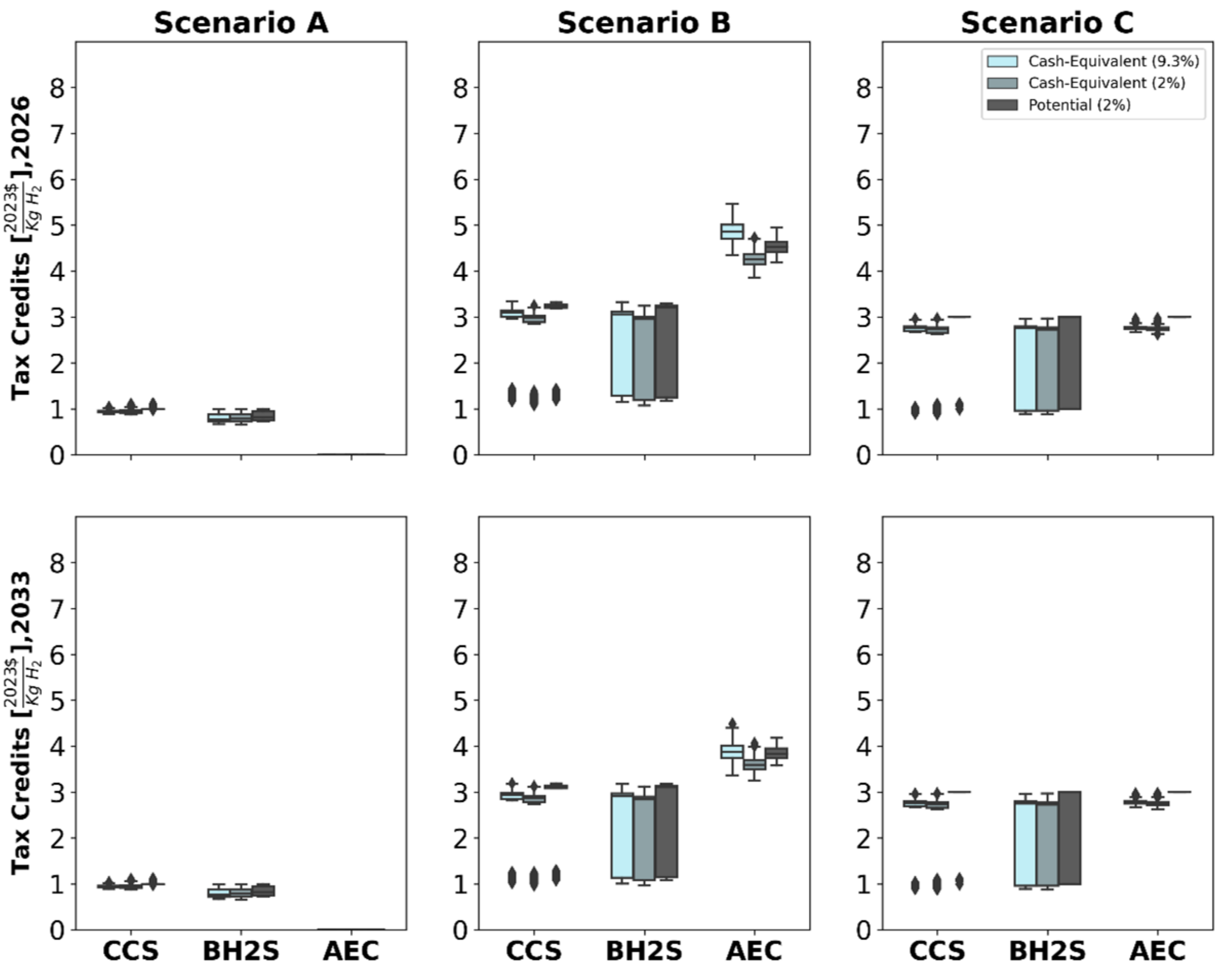

Supplementary Figure 7: Yearly matched total policy support for ammonia technologies. This figure compares the total policy support (in \$ per kg H_2) provided for various low-carbon ammonia production technologies across two main scenarios—Scenario A and Scenarios B/C—and two different electricity configurations: grid-based electricity and hybrid renewable electricity farms. Scenario A accounts for policy support related to emissions from the stack, feedstock, and electricity sources, while Scenarios B and C assume policy support for stack and feedstock emissions only, under the assumption of zero emissions from renewable energy sources. CCS refers to ammonia production with carbon capture, BH2S refers to ammonia production with biomass gasification, and AEC refers to ammonia production through alkaline electrolysis. The light blue boxes signify cash-equivalent tax credits discounted at 9.8%, which are credits adjusted for income tax. The darker blue boxes represent the same tax credits discounted at the risk-free rate, while the gray boxes denote the same tax credits without accounting for income tax.

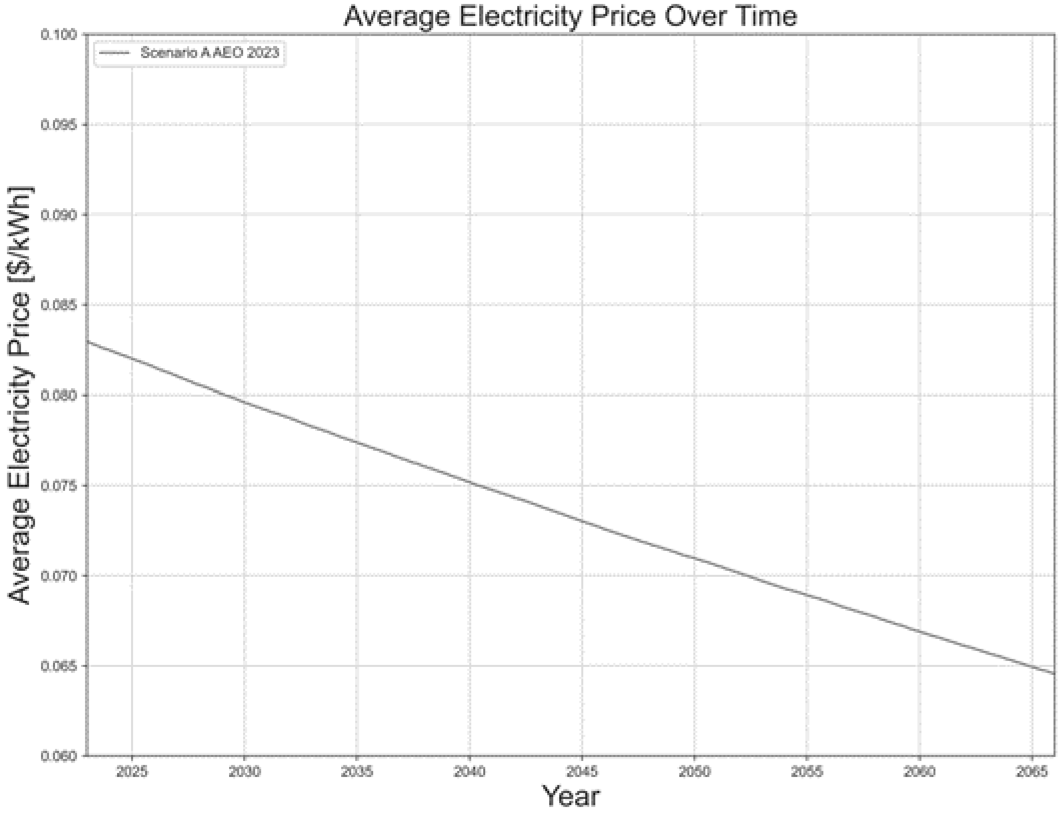

Supplementary Figure 8: Mean electricity price over time. The GBM (geometric brownian motion) model collapses to a linear model across 4000 simulations.

Supplementary Tables

Supplementary Table 37: Comparison of inputs of IEA versus this study.

**Inputs**	**IEA, 2021**	**This study**
Electricity (cents per kWh)	1.0 – 10	6.3
Natural gas ($ per MMBtu)	2.8 – 7.765	2
Availability (%time)	95	90
Discount factor (%$)	8	9.3
CAPEX heuristics (%PEC)[a]	+70	+100
Lifetime (years)	25	40

Abbreviations: IEA, International Energy Agency; CAPEX, capital expenditure; PEC, purchased equipment cost; MMBtu, million British thermal units; kWh, kilowatt-hour. The table compares key input parameters between the IEA (2021) study and this study, highlighting differences in assumptions such as electricity costs, natural gas prices, availability, discount factors, CAPEX heuristics, and project lifetime.

Supplementary References

[1] Abdukarem Amhamed, Abdulkarem Amhamed, Syed Shuibul Qarnain, Sally Hewlett, Ahmed Sodiq, Yasser Abdellatif, Rima J Isaifan, and Odi Fawwaz Alrebei. Ammonia production plants—a review. *Fuels*, 2022.

[2] Eric Lewis, Shannon McNaul, Matthew Jamieson, Megan Henriksen, H Scott Matthews, Liam Walsh, Jadon Grove, Travis Shultz, and Robert Stevens. Comparison of commercial, state-of-the-art, fossil-based hydrogen production technologies. *null*, 2022.

[3] André F Young, André F Young, Hugo Gomes D'Amato Villardi, Hugo G D Villardi, Leonardo Sales Araújo, Luciano Santos Constantin Raptopoulos, and Max Suell Dutra. Detailed design and economic evaluation of a cryogenic air separation unit with recent literature solutions. *Industrial Engineering Chemistry Research*, 2021.

[4] Robert D Brasington, John L Haslback, Norma Kuehn, Eric Lewis, Lora L Pinkerton, Marc J Turner, Elsy Varghese, and Mark Woods. Cost and performance baseline for fossil energy plants - volume 2: Coal to synthetic natural gas and ammonia. *null*, 2011.

[5] Guido Collodi, Giuliana Azzaro, and Noemi Ferrari. Techno-economic evaluation for smr base standalone (merchant) plant with ccs, 2 2017.

[6] P Spath, Andy Aden, T Eggeman, M Ringer, B Wallace, and J Jechura. Biomass to hydrogen production detailed design and economics utilizing the battelle columbus laboratory indirectly-heated gasifier. 2005.

[7] Hans Böhm, Andreas Zauner, Daniel C Rosenfeld, and Robert Tichler. Projecting cost development for future large-scale power-to-gas implementations by scaling effects. *Applied Energy*, 2020.

[8] Oliver Schmidt, Ajay Gambhir, Iain Staffell, Adam Hawkes, Jenny Nelson, and Sheridan Few. Future cost and performance of water electrolysis: An expert elicitation study. *International Journal of Hydrogen Energy*, 2017.

[9] Joana Sousa, Wendelin Waiblinger, and K Andreas Friedrich. Techno-economic study of an electrolysis-based green ammonia production plant. *Industrial Engineering Chemistry Research*, 2022.

[10] R Turton, J Shaeiwitz, D Bhattacharyya, and W Whiting. *Analysis, Synthesis, and Design of Chemical Processes*. Prentice Hall, 5 edition, 2018.

[11] Jörn Brauns and Thomas Turek. Alkaline water electrolysis powered by renewable energy: A review, 2020.

[12] Max Peters, Klaus Timmerhaus, and Ronald West. *Plant Design and Economics for Chemical Engineers*. McGraw Hill, New York, 5th edition, 2002.

[13] Emanuele Taibi, Herib Blanco, Raul Miranda, Marcelo Carmo, Dolf Gielen, and Roland Roesch. Green hydrogen cost reduction - scaling up electrolysers to meet the 1.5°c climate goal, 2020.

[14] U.S. Energy Information Administration. Annual energy outlook 2023, 2023. Accessed: 2024-08-10.

[15] Patrick R. O'Donoughue, Garvin A. Heath, Stacey L. Dolan, and Martin Vorum. Life cycle greenhouse gas emissions of electricity generated from conventionally produced natural gas. *Journal of Industrial Ecology*, 18:125–144, 2 2014.

[16] EPA. Supplementary material for the regulatory impact analysis for the supplemental proposed rulemaking, "standards of performance for new, reconstructed, and modified sources and emissions guidelines for existing sources: Oil and natural gas sector climate review", 9 2022.

[17] Andrea Ben-Yosef. Irs issues proposed rules on direct-pay elections of applicable energy tax credits, 6 2023.

[18] Rachel Chang. Understanding direct pay and transferability for tax credits in the inflation reduction ac, 6 2023.

[19] C McKenna, N Knapp, B von Oesen, G Durden, M Hebbar, J Manning, M Tatarsky, and M Yurkerwich. Inflation reduction act: Tax credit monetization analysis, 8 2022.

[20] Renewable energy incentives from the inflation reduction act, 11 2022.

[21] Scott Nicholson and Gavin Heath. Life cycle emissions factors for electricity generation technologies, 2021.

[22] Aswath Damodaran. Cost of equity and capital (us), 1 2023.

[23] U.S. Congress. Inflation reduction act (ira), 2022. Pub. L. No.117-169, 136 Stat. 1818.

[24] Stefan Pfenninger and Iain Staffell. Long-term patterns of european pv output using 30 years of validated hourly reanalysis and satellite data. *Energy*, 114:1251–1265, 11 2016.

[25] Open Energy Data Initiative (OEDI). Oedi: 2022 annual technology baseline (atb) cost and performance data for electricity generation technologies, 2022.

[26] John Bistline, Geoffrey Blanford, Maxwell Brown, Dallas Burtraw, Maya Domeshek, Jamil Farbes, Allen Fawcett, Anne Hamilton, Jesse Jenkins, Ryan Jones, Ben King, Hannah Kolus, John Larsen, Amanda Levin, Megan Mahajan, Cara Marcy, Erin Mayfield, James McFarland, Haewon McJeon, Robbie Orvis, Neha Patankar, Kevin Rennert, Christopher Roney, Nicholas Roy, Greg Schivley, Daniel Steinberg, Nadejda Victor, Shelley Wenzel, John Weyant, Ryan Wiser, Mei Yuan, and Alicia Zhao. Emissions and energy impacts of the inflation reduction act. *Science*, 380:1324–1327, 6 2023.

[27] Tyler Stehly, Philipp Beiter, and Patrick Duffy. 2019 cost of wind energy review. 2020.

[28] Wesley J Cole and Allister Frazier. Cost projections for utility-scale battery storage. 2019.

[29] Jeff McDonald. Cbam, ets reform to impact fertilizer trade. *SP Global Commodity Insights*, 2 2023.

[30] G. Kakoulaki, I. Kougias, N. Taylor, F. Dolci, J. Moya, and A. Jäger-Waldau. Green hydrogen in europe – a regional assessment: Substituting existing production with electrolysis powered by renewables. *Energy Conversion and Management*, 228, 2021.

[31] European Commission. Communication com/2020/301: A hydrogen strategy for a climate-neutral europe, 2020. Accessed: 2024-08-10.

[32] U.s. ammonia production is growing, and becoming less carbon intensive. *Energy Information Administration*, 4 2021.

[33] Max Appl. *Process Steps of Ammonia Production*, pages 65–176. 3 1999. Wiley Online Books.

[34] Kevin Rouwenhorst, Gabriel Castellanos, Francisco Boshell, Roland Roesch, Dolf Gielen, Trevor Brown, Emanuele Taibi, Herib Blanco, Barbara Jinks, Emanuele Bianco, and Ufuk Sezer. Innovation outlook: Renewable ammonia, 2022.

[35] Douglas R MacFarlane, Pavel V Cherepanov, Jaecheol Choi, Bryan H R Suryanto, Rebecca Y Hodgetts, Jacinta M Bakker, Federico M Ferrero Vallana, and Alexandr N Simonov. A roadmap to the ammonia economy. *Joule*, 4:1186–1205, 2020.

[36] Sara Budinis, Alexandre Gouy, Peter Levi, Hana Mandová, Tiffany Vass, Araceli Fernández Pales, and Timur Gül. Ammonia technology roadmap: Towards more sustainable nitrogen fertilizer production. *International Energy Agency*, 2021.

[37] Gary R Maxwell. Synthetic nitrogen products. 2012.

[38] Hanfei Zhang, Hanfei Zhang, Ligang Wang, Jan Van herle, François Maréchal, and Umberto Desideri. Techno-economic comparison of green ammonia production processes. *Applied Energy*, 2020.

[39] Pratham Arora, Andrew Hoadley, Sanjay M Mahajani, and Anuradda Ganesh. Multi-objective optimization of biomass based ammonia production - potential and perspective in different countries. *Journal of Cleaner Production*, 2017.

[40] Laurence Tock, François Maréchal, and Matthieu Perrenoud. Thermo-environomic evaluation of the ammonia production. *null*, 2013.

[41] Per Tunå, Christian Hulteberg, and Serina Ahlgren. Techno-economic assessment of nonfossil ammonia production. *Environmental Progress*, 2014.

[42] Kenia Gabriela dos Santos, Caroline Thaís Eckert, Eduardo de Rossi, Reinaldo Aparecido Bariccatti, Elisandro Pires Frigo, Elisandro Pires Frigo, Cleber Antonio Lindino, Cleber Antonio Lindino, and Helton José Alves. Hydrogen production in the electrolysis of water in brazil, a review. *Renewable Sustainable Energy Reviews*, 2017.

[43] Joris Proost. State-of-the-art capex data for water electrolysers, and their impact on renewable hydrogen price settings. *International journal of hydrogen energy*, 2017.

[44] Boreum Lee, Dongjun Lim, Hyunjun Lee, and Hankwon Lim. Which water electrolysis technology is appropriate?: Critical insights of potential water electrolysis for green ammonia production. *Renewable Sustainable Energy Reviews*, 2021.

[45] Surinder P. Singh, Anthony Y. Ku, Niall Macdowell, and Can Cao. Profitability and the use of flexible co2 capture and storage (ccs) in the transition to decarbonized electricity systems. *International Journal of Greenhouse Gas Control*, 120:103767, 10 2022.

[46] Jamie Gomez, John M Baca, Fernando H Garzon, and Fernando H Garzon. Techno-economic analysis and life cycle assessment for electrochemical ammonia production using proton conducting membrane. *International Journal of Hydrogen Energy*, 2020.

[47] Andrew M Simons, Andrew Simons, and Christian Bauer. Life cycle assessment of hydrogen production. *null*, 2011.

[48] Pratham Arora, Andrew Hoadley, Sanjay M Mahajani, and Anuradda Ganesh. Small-scale ammonia production from biomass: A techno-enviro-economic perspective. *Industrial Engineering Chemistry Research*, 2016.

[49] Nicolas Campion, Hossein Nami, Philip Swisher, Peter Vang Hendriksen, and Marie Münster. Techno-economic assessment of green ammonia production with different wind and solar potentials. *Social Science Research Network*, 2022.

[50] Carlos Arnaiz del Pozo and Schalk Cloete. Techno-economic assessment of blue and green ammonia as energy carriers in a low-carbon future. *Energy Conversion and Management*, 255:115312, 2022.

[51] C Fúnez Guerra, Lorenzo Reyes-Bozo, Eduardo Vyhmeister, M Jaén Caparrós, José Luis Salazar, José Luis Salazar, and Carmen Clemente-Jul. Technical-economic analysis for a green ammonia production plant in chile and its subsequent transport to japan. *Renewable Energy*, 2020.

[52] Ola Osman, Sgouris Sgouridis, and Andrei Sleptchenko. Scaling the production of renewable ammonia: A techno-economic optimization applied in regions with high insolation. *Journal of Cleaner Production*, 2020.

[53] Kevin H.R. Rouwenhorst, Aloijsius G.J. Van der Ham, Guido Mul, and Sascha R.A. Kersten. Islanded ammonia power systems: Technology review conceptual process design, 10 2019.

[54] Antonio Sánchez, Antonio Sánchez, Mariano Martín, and Pastora Vega. Biomass based sustainable ammonia production: Digestion vs gasification. *ACS Sustainable Chemistry Engineering*, 2019.

[55] Ganzhou Wang, Alexander Mitsos, and Wolfgang Marquardt. Renewable production of ammonia and nitric acid. *Aiche Journal*, 2020.

[56] Jesse Jenkins and Wilson Ricks. The cost of clean hydrogen with robust emissions standards: A comparison across studies, 4 2023.

[57] Collin Smith, Alfred K Hill, and Laura Torrente-Murciano. Current and future role of haber–bosch ammonia in a carbon-free energy landscape. *Energy and Environmental Science*, 2020.

[58] Peter H Pfromm. Towards sustainable agriculture: Fossil-free ammonia. *Journal of Renewable and Sustainable Energy*, 2017.

[59] Yusuf Bicer, Ibrahim Dincer, Calin Zamfirescu, Greg Vezina, and Frank Raso. Comparative life cycle assessment of various ammonia production methods. *Journal of Cleaner Production*, 2016.

[60] Yusuf Bicer and Ibrahim Dincer. Life cycle assessment of nuclear-based hydrogen and ammonia production options: A comparative evaluation. *International Journal of Hydrogen Energy*, 2017.

[61] Xinyu Liu, Amgad Elgowainy, Michael Wang, and Michael Wang. Life cycle energy use and greenhouse gas emissions of ammonia production from renewable resources and industrial by-products. *Green Chemistry*, 2020.

[62] Ben Young, Michelle Krynock, Derrick R Carlson, Troy Hawkins, Troy Hawkins, Troy R Hawkins, Joe Marriott, Ben Morelli, Matthew Jamieson, Matt Jamieson, Gregory Cooney, Greg Cooney, Timothy J Skone, Timothy J Skone, and Timothy J Skone. Comparative environmental life cycle assessment of carbon capture for petroleum refining, ammonia production, and thermoelectric power generation in the united states. *International Journal of Greenhouse Gas Control*, 2019.

[63] Christian Bauer, Karin Treyer, Cristina Antonini, Joule Bergerson, Matteo Gazzani, Emre Gencer, Jon Gibbins, Marco Mazzotti, Sean T. McCoy, Russell McKenna, Robert Pietzcker, Arvind P. Ravikumar, Matteo C. Romano, Falko Ueckerdt, Jaap Vente, and Mijndert van der Spek. On the climate impacts of blue hydrogen production. *Sustainable Energy and Fuels*, 6, 2022.

[64] Paul Gilbert, Sarah Alexander, Patricia Thornley, and John Brammer. Assessing economically viable carbon reductions for the production of ammonia from biomass gasification. *Journal of Cleaner Production*, 2014.

[65] Abhijeet P Borole and Anne Landfield Greig. Life-cycle assessment and systems analysis of hydrogen production. *Biohydrogen*, 2019.

[66] Antonio Valente, Diego Iribarren, and Javier Dufour. Prospective carbon footprint comparison of hydrogen options. *Science of The Total Environment*, 2020.

[67] R Ostundi and F Zardi. Method for load regulation of an ammonia plant, 2011.

[68] Kevin Verleysen, Alessandro Parente, and Francesco Contino. How does a resilient, flexible ammonia process look? robust design optimization of a haber-bosch process with optimal dynamic control powered by wind. *Proceedings of the Combustion Institute*, 39, 2023.

[69] Izzat Iqbal Cheema and Ulrike Krewer. Operating envelope of haber-bosch process design for power-to-ammonia. *RSC Advances*, 8, 2018.

[70] Kamyll Dawn Cocon. *Towards an agile Power-to-Ammonia pathway: optimization of a dynamic ammonia production process*. PhD thesis, Ecole polytechnique de Louvain, Université catholique de Louvain, 2021. Prom.: Contino, Francesco.

[71] Julien Armijo and Cédric Philibert. Flexible production of green hydrogen and ammonia from variable solar and wind energy: Case study of chile and argentina. *International Journal of Hydrogen Energy*, 2020.

[72] Changlong Wang, Stuart D.C. Walsh, Thomas Longden, Graham Palmer, Israel Lutalo, and Roger Dargaville. Optimising renewable generation configurations of off-grid green ammonia production systems considering haber-bosch flexibility. *Energy Conversion and Management*, 280:116790, 3 2023.

[73] Nikifar Lazouski, Aditya Limaye, Abhishek Bose, Michal L. Gala, Karthish Manthiram, and Dharik S. Mallapragada. Cost and performance targets for fully electrochemical ammonia production under flexible operation. *ACS Energy Letters*, 7:2627–2633, 8 2022.

[74] Hiroaki Onodera, Rémi Delage, and Toshihiko Nakata. Systematic effects of flexible power-to-x operation in a renewable energy system - a case study from japan. *Energy Conversion and Management: X*, 20:100416, 10 2023.

[75] Abhishek Bose, Nikifar Lazouski, Michal L. Gala, Karthish Manthiram, and Dharik S. Mallapragada. Spatial variation in cost of electricity-driven continuous ammonia production in the united states. *ACS Sustainable Chemistry Engineering*, 10:7862–7872, 6 2022.

[76] Houssam Bouaboula, Mohammed Ouikhalfan, Ismael Saadoune, Jamal Chaouki, Abdelghafour Zaabout, and Youssef Belmabkhout. Addressing sustainable energy intermittence for green ammonia production. *Energy Reports*, 9:4507–4517, 12 2023.

[77] T Davenne, A Huddart, R Cowan, D Tallentire, B Peters, J Smith, A Ruddell, and J Halliday. Ammonia synthesis plant from intermittent renewable energy (aspire). frazer-nash consultancy., 2022.

[78] K Rouwenhorst. China: Scaling-up “flexible” ammonia production powered by renewable energy. *Ammonia Energy Association*, 2023.
